# NCIVISION: A Siamese Neural Network for Molecular Similarity Prediction MEP and RDG Images

**DOI:** 10.3390/molecules30234589

**Published:** 2025-11-28

**Authors:** Rafael Campos Vieira, Letícia de A. Nascimento, Arthur Alves Nascimento, Nicolas Ricardo de Melo Alves, Érica C. M. Nascimento, João B. L. Martins

**Affiliations:** 1 Department of Pharmacy, Faculty of Health Sciences, University of Brasilia, Brasilia 70910-900, DF, Brazil; rafaelcamposunb@gmail.com (R.C.V.); leticiaalm2000@gmail.com (L.d.A.N.); 2 Laboratory of Computational Chemistry, Institute of Chemistry, University of Brasilia, Brasilia 70910-900, DF, Brazil; ziontemplar@gmail.com (A.A.N.); nicolasrmalves@gmail.com (N.R.d.M.A.); ericacristinamoreno@gmail.com (É.C.M.N.)

**Keywords:** convolutional neural networks, embeddings, Siamese neural network, similarity, MEP, RDG

## Abstract

Artificial neural networks in drug discovery have shown remarkable potential in various areas, including molecular similarity assessment and virtual screening. This study presents a novel multimodal Siamese neural network architecture. The aim was to join molecular electrostatic potential (MEP) images with the texture features derived from reduced density gradient (RDG) diagrams for enhanced molecular similarity prediction. On one side, the proposed model is combined with a convolutional neural network (CNN) for processing MEP visual information. This data is added to the multilayer perceptron (MLP) that extracts texture features from gray-level co-occurrence matrices (GLCM) computed from RDG diagrams. Both representations converge through a multimodal projector into a shared embedding space, which was trained using triplet loss to learn similarity and dissimilarity patterns. Limitations associated with the use of purely structural descriptors were overcome by incorporating non-covalent interaction information through RDG profiles, which enables the identification of bioisosteric relationships needed for rational drug design. Three datasets were used to evaluate the performance of the developed model: tyrosine kinase inhibitors (TKIs) targeting the mutant T315I BCR-ABL receptor for the treatment of chronic myeloid leukemia, acetylcholinesterase inhibitors (AChEIs) for Alzheimer’s disease therapy, and heterodimeric AChEI candidates for cross-validation. The visual and texture features of the Siamese architecture help in the capture of molecular similarities based on electrostatic and non-covalent interaction profiles. Therefore, the developed protocol offers a suitable approach in computational drug discovery, being a promising framework for virtual screening, drug repositioning, and the identification of novel therapeutic candidates.

## 1. Introduction

The use of artificial neural networks (ANNs) in chemistry has established itself as a field of intense research. The applications go beyond learning chemical syntax through language models (LLMs). They also include autoencoders for generating new molecules using SMILES representations or three-dimensional molecular structure data [1,2,3]. Furthermore, ANNs have been widely employed in predicting physicochemical properties, which are relevant to the development of new materials and pharmaceuticals [4,5].

Among the various ANN architectures, convolutional neural networks (CNNs) stand out, particularly in tasks involving image-format data. This performance is due to convolutional operations that enable the network to extract and recognize visual patterns, allowing applications in classification, similarity assessment, and pattern recognition problems.

In chemistry, CNNs have been applied in multiple areas, including the prediction of chemical reactivity and the quantitative estimation of polymer properties [6,7]. In terms of drug design, these networks have shown promise in tasks such as predicting protein-ligand binding affinities in docking simulations [8], virtual screening [9], and ligand poses within protein active sites [10], as well as in determining pharmacological properties such as solubility and lipophilicity [11] in the design of novel molecular entities [12].

Large-scale models, such as CNN-Drug-Drug Interaction (CNN-DDI) [13] and the Hybrid Convolutional Neural Network (HCNN) [14], incorporate similarity metrics (such as the Jaccard similarity) used to enhance predictive performance regarding drug-drug interactions. These metrics are fundamental in drug design, as they assist in both the discovery of new drug candidates and in the repositioning of existing drugs, thereby optimizing the virtual screening process. Therefore, the development of models capable of accurately identifying similarities and dissimilarities between compounds is essential for advancing this field.

In the context of molecular similarity among drug candidates, it is important to note that using only structural descriptors is insufficient to reveal similarity between molecules. Non-covalent interactions, including hydrogen bonding, electrostatic interactions, and van der Waals forces, play a central role in the stability of protein-ligand complexes. Molecules that are grouped together but have different structures can exhibit similar molecular electrostatic potential (MEP) profiles. These profiles represent the three-dimensional distribution of electrostatic potential around a molecule. This trend results in similar interactions with the active site of the target protein and, consequently, similar biological activity. Such molecular groups are known as bioisosteres and play a crucial role in rational drug design and molecular repositioning on other bioreceptor targets.

Siamese neural networks (SNNs) have provided suitable applications to drug design and development [9,15,16,17]. The present study aims to develop a model based on SNN, using as input MEP images and gray-level co-occurrence matrices (GLCM) extracted from reduced density gradient (RDG) diagrams. RDG diagrams are a quantum chemical method, particularly important for visualizing non-covalent interactions, such as van der Waals interactions and hydrogen bonds, by plotting the reduced density gradient as a function of the electron density, thereby enabling the identification and characterization of intermolecular interaction regions [18,19]. On one side, MEP images are processed following the CNN approach, while the GLCM matrices are converted into feature vectors via a multilayer perceptron (MLP) integrated into the model. We have studied two well-known biological targets: the mutant tyrosine kinase T315I BCR-ABL inhibition, which is the main bioreceptor target for treating Chronic Myeloid Leukemia (CML) [20], and the human acetylcholinesterase (hAChE) inhibition, currently one of the main approaches to treating deleterious cognitive side effects of Alzheimer’s disease [21]. We have used different datasets to evaluate the model’s ability to predict ligand similarity based on visual data. The first dataset comprised tyrosine kinase inhibitors (TKIs) [22,23,24], while the second involved acetylcholinesterase inhibitors (AChEIs) candidates, using donepezil (DNP) as a negative control to test the model’s robustness with low-similarity triplets. The third dataset, also composed of potential AChEIs designed as heterodimers, was used for cross-validation to compare the performance of models trained on the two different datasets. This study is expected to expand the applicability of CNNs in molecular similarity analysis within the identical context of drug development.

## 2. Results and Discussion

Our training procedure used the known early stopping approach, which prevents overfitting and provides optimal model generalization. This approach is a standard technique in deep learning, where training is terminated when validation performance plateaus or begins to deteriorate. The 20-epoch duration represents the optimal length for the datasets under investigation.

**Figure 1:**
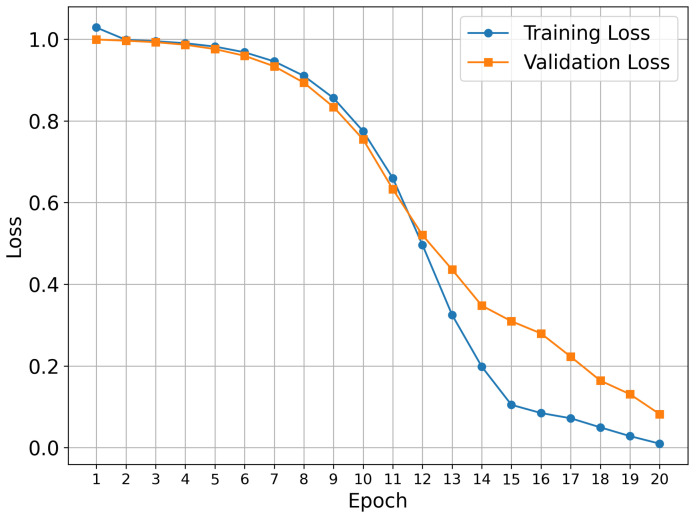
Model performance during training and validation for the TKIs dataset.

Figure 1 shows the training results for the TKIs dataset. The training process demonstrates proper convergence, with validation and training curves closely aligned and the loss approaching zero in the final epochs. For this dataset, the model focused its attention on the most electropositive region of ponatinib, specifically on the piperazine moiety. Although Figure 1 showed a continued decline at epoch 20, suggesting that the equilibrium stage had not been achieved, the early stopping technique was triggered by validation metrics rather than visual inspection alone. This approach prioritizes robust statistical indicators over potentially misleading visual patterns, resulting in a stable performance and convergence within the 20-epoch framework.

We examined Grad-CAM visualizations [25] to assess each model’s ability to detect spatial modifications and interpret its decision process. Grad-CAM (Gradient-weighted Class Activation Mapping) highlights class-discriminative regions of molecular structures that drive a prediction. This method generates heatmaps that encode relevance, where red denotes a strong contribution to the decision and blue indicates a weak contribution.

It is important to note that in all our Grad-CAM heatmap visualizations, the colorscale is independent of the original MEP colorscale. Although MEP images contain their own color-coded electrostatic potential values, the Grad-CAM overlay represents a separate attention-based metric that indicates which spatial regions the trained model prioritizes during the classification process. The model learns from the MEP color patterns, textures, and transitions via GLCM features. However, the resulting Grad-CAM visualization uses its own normalized scale (0.0–1.0) to represent attention intensity rather than electrostatic potential magnitude.

**Figure 2:**
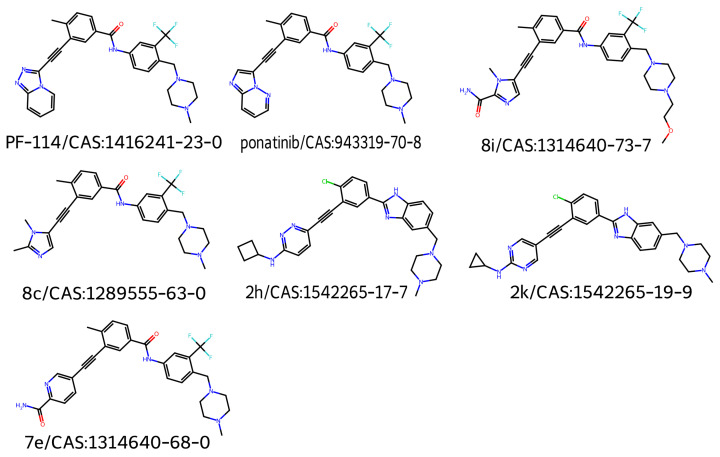
2D structures for major molecules for TKIs dataset.

**Figure 3:**
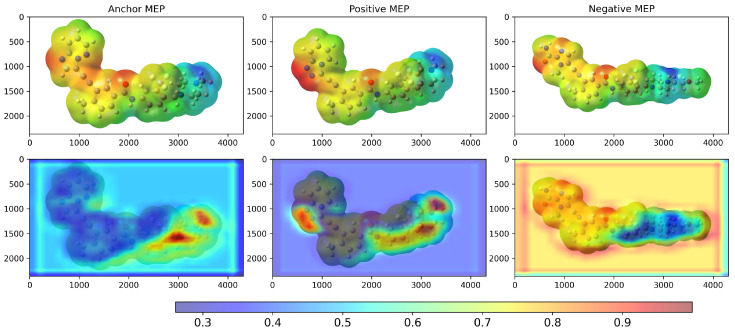
Grad-CAM result of the model after training on the TKI molecules dataset. The X and Y coordinates represent the width and height, respectively, of the original MEP images in pixel units. The colorbar scale represents the normalized activation intensity (0.0–1.0), where higher values indicate regions that receive more attention from the model during classification. Anchor = ponatinib, Positive = PF-114, Negative = 8i.

Figure 2 shows the 2D structures of the major molecules in the TKIs dataset. As shown in Figure 3, the model correctly identified the piperazine-containing region of the compound PF-114 [26] as chemically analogous to the ponatinib anchor in the three-dimensional chemical feature space. The model also accurately recognized the modified purine cores of PF-114 ([1,2,4]triazolo[4,3-a]pyridine ring) and ponatinib (imidazo[1,2-b]pyridazine ring) as structurally similar.

**Figure 4:**
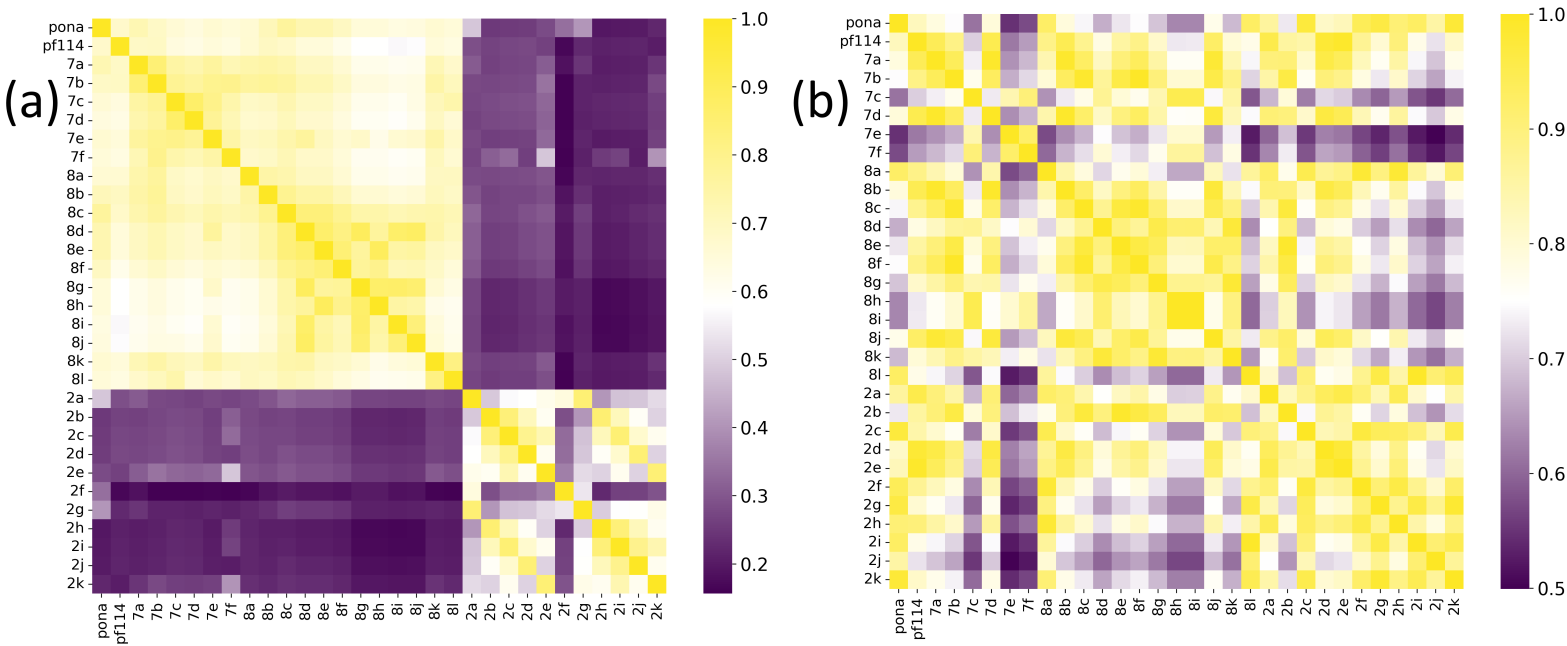
(**a**) Tanimoto similarity using SMILES, and (**b**) model-based similarity (via inverse distance). Data from TKI molecules. The acronyms for ponatinib (labeled pona in the image) and PF-114 (labeled pf114 in the image) were used.

Figure 3 also suggests that the model was capable of identifying spatial modifications in terms of dissimilarity. An example of this behavior is shown in Figure 4, which compares pairwise molecular similarity computed using Tanimoto coefficients with model-derived inverted distances in the learned space. Compounds such as 8i, which have SMILES strings similar to ponatinib (left panel), are located far from ponatinib in the model’s learned space (right panel). Despite having a similar atomic composition to the purine-like core of ponatinib, Figure 3 shows that the model focuses on the imidazole group in compound 8i. The possible explanation is that the imidazole group of compound 8i does not induce the same steric strain as the purine-like core of ponatinib does.

Another relevant factor is that the model outperformed the heuristic approach, since the input triplets were defined based on Tanimoto similarity scores. This behavior demonstrates that the model was able to capture not only physicochemical features but also spatial and electrostatic characteristics—reflected in the MEP maps and RDG diagrams—representing an association, albeit indirect, with the types of non-covalent interactions present in the molecules.

**Figure 5:**
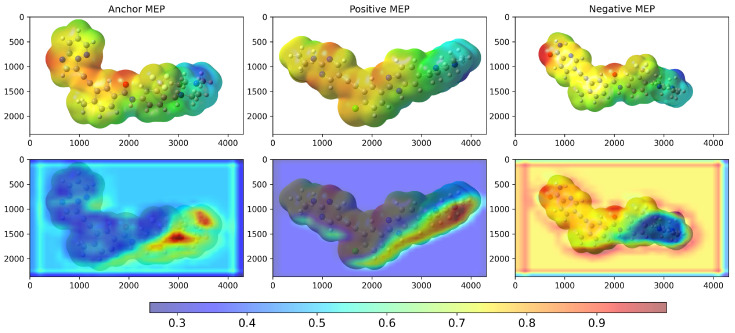
Grad-CAM (**bottom**) result of the model after training on the TKI molecules dataset, considering the highest and lowest $d^{-1}$ values computed by the model. The X and Y coordinates represent the width and height, respectively, of the original MEP (**top**) images in pixel units. The colorbar scale represents the normalized activation intensity (0.0–1.0), where higher values indicate regions that receive more attention from the model during classification. The anchor value is ponatinib, the positive control is 2k, and the negative control is 7e.

In line with this feature, Figure 5 indicates that the highest- and lowest-scoring compounds classified as positive and negative by the model are directly associated with the shape pattern of the electrostatic potential surface around the molecule, for both similarity and dissimilarity. Therefore, the inversion in similarity ranking for several molecules occurs when comparing Tanimoto similarity via SMILES (using RDKit) to the inverted distance computed by the model.

Compound 8c exhibited the highest Tanimoto similarity score ($S_{T}=0.776$), achieving a similar inverse distance value as calculated by the model ($d^{-1}=0.741$). However, notable discrepancies were observed in the similarity predictions for compounds from Class 2. The Tanimoto similarity between these compounds and ponatinib was low. Compound 2h had the lowest similarity among all TKI molecule class compounds, with a value of $S_{T}=0.195$. The model calculations showed that these compounds had the highest inverse distance values, with compound 2k having the highest value ($d^{-1}=0.978$).

**Figure 6:**
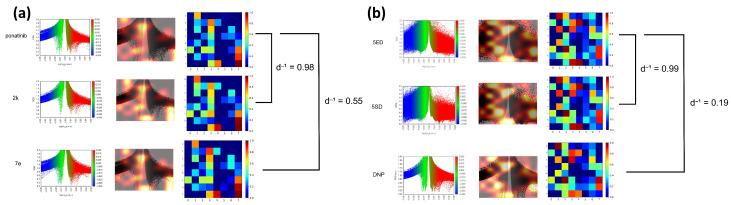
(**a**) Saliency map and embedding projection for the molecule closest (2k, $d^{-1}=0.98$) and farthest (7e, $d^{-1}=0.55$) from ponatinib. (**b**) Saliency map and embedding projection for the molecule closest (5SD, $d^{-1}=0.99$) and farthest (DNP, $d^{-1}=0.19$) from 5ED.

**Figure 7:**
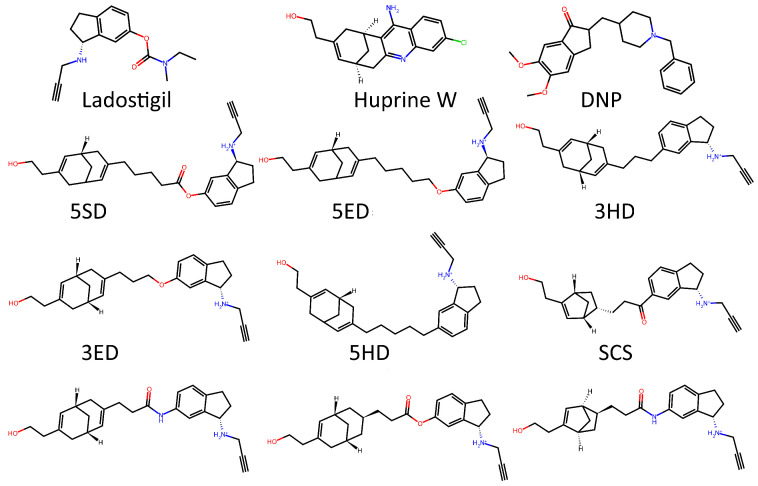
2D structures for major molecules for AChEIs based huprine-ladostigil dataset.

Conversely, the compound with the lowest inverse distance was 7e ($d^{-1}=0.547$), which showed an intermediate Tanimoto similarity value ($S_{T}=0.666$). Figure 6 shows saliency maps over RDG diagrams together with the corresponding embedding projections. Figure 7a shows the molecules closest (2k) and farthest (7e) from ponatinib. Figure 7b presents the molecules closest (5SD) and farthest (DNP) from 5ED. In both cases, the model highlights informative regions of the RDG diagrams, exhibits focused attention on RDG peaks and edges, reflecting the texture sensitivity of the GLCM-based input, and demonstrates the effectiveness of GLCM matrices for similarity analysis. Note that these maps reflect only the RDG/GLCM branch; MEP images yield the other input stream and provide complementary electrostatic information for similarity learning.

**Figure 8:**
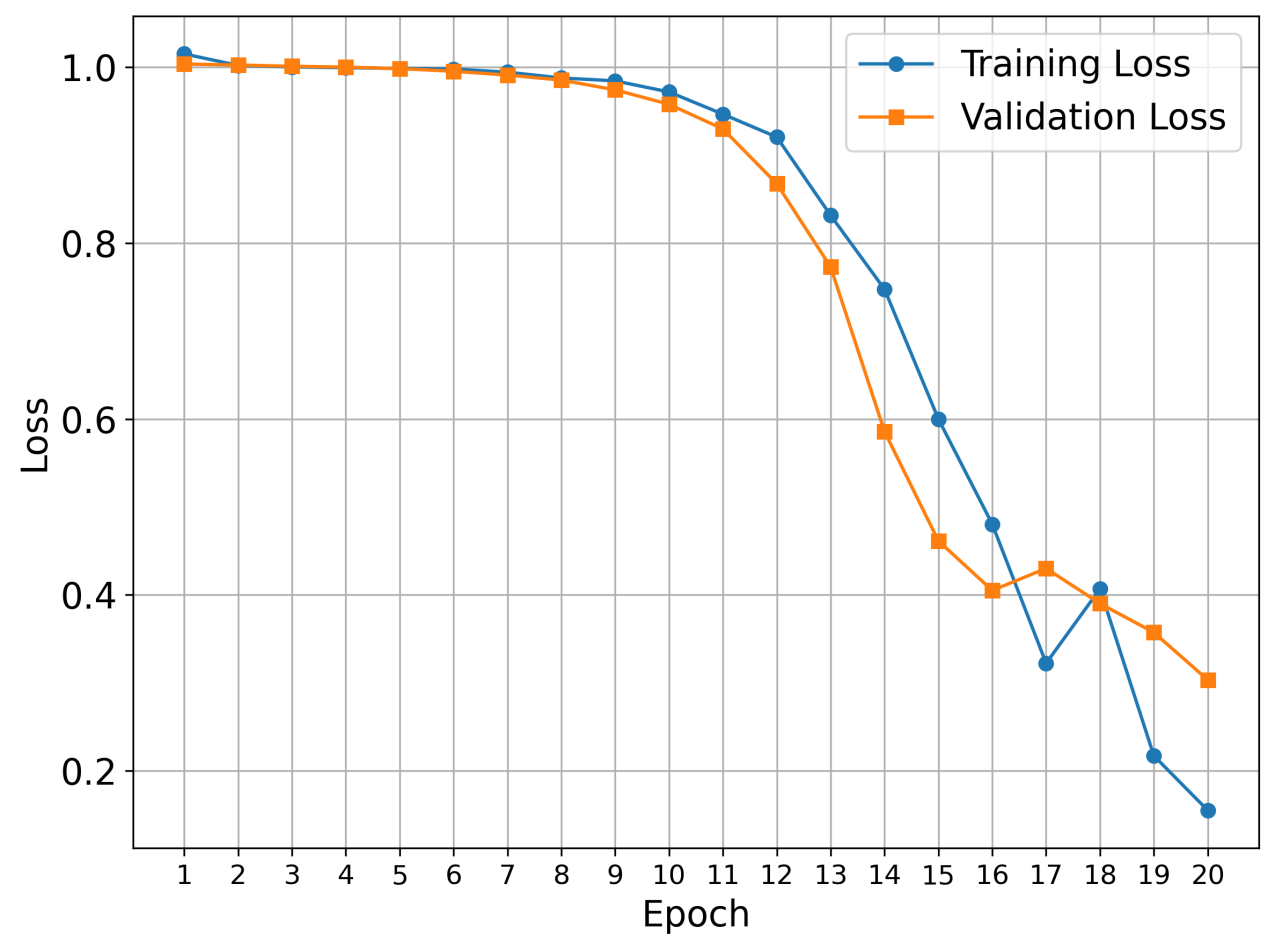
Model performance during training and validation on the potential AChEI dataset.

To test whether the model was truly capable of identifying shape patterns independently of the predefined triplets, as well as to assess its learning capacity, we employed a second dataset. This dataset consisted of 31 candidate molecules for AChE inhibition (AChEI), theorized by our research group, using huprine W and ladostigil as starting points. Figure 7 shows the 2D structures of the major molecules for this dataset. All triplets formed in this dataset can be considered hard triplets since we used donepezil as the anchor. Therefore, all resulting triplets exhibited low similarity to DNP. For triplet formation, compounds were defined as positives if they had a Tanimoto similarity score of $S_{T}\geq 0.13$, and as negatives if $S_{T}\leq 0.12$. The effect of hard triplets during model training can be observed in Figure 8, where a bottleneck is evident in both training and validation curves during the final epochs, representing the increased difficulty for the model to converge in minimizing the triplet loss.

The highest inverse distance value, $d^{-1}$, predicted by the model was observed between the pair 5ED and 5SD ($d^{-1}=0.994$), while the lowest value was found between 5ED and DNP ($d^{-1}=0.186$). These GLCM-based embeddings can be visualized in Figure 6. Regarding Tanimoto similarity ($S_{T}$), the highest value was obtained for the pair 3HD–5HD ($S_{T}=0.944$). This same pair showed a model-based inverse distance of $d^{-1}=0.667$. In contrast, the 5ED–5SD pair had a Tanimoto similarity of $S_{T}=0.761$, while the highest Tanimoto similarity for 5ED was with 3ED ($S_{T}=0.931$).

**Figure 9:**
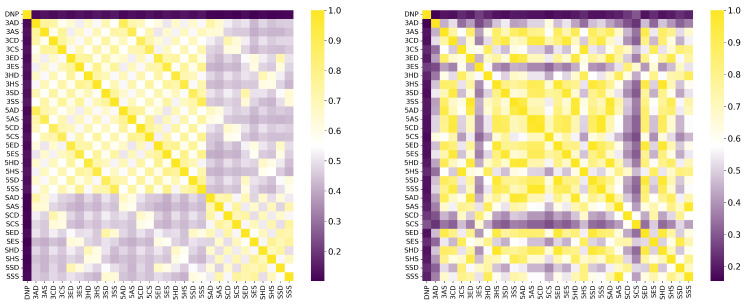
Tanimoto similarity using SMILES (**left**) and model-based similarity (via inverse distance) (**right**). Data from AChEI candidates using DNP as the negative control.

Figure 9 illustrates how the model maintained low similarity scores with the control compound DNP, while simultaneously identifying spatial similarity patterns in the MEP images, as captured by the GLCM feature vectors. This feature resulted in the expected identification of similarity between chemical structures, as pointed out by the high similarity observed between 5ED and 5HD, and the reduction in similarity between 3HD and 5HD. Despite being structurally similar, they exhibit considerable spatial differences, leading to distinct electrostatic potential interaction profiles.

**Figure 10:**
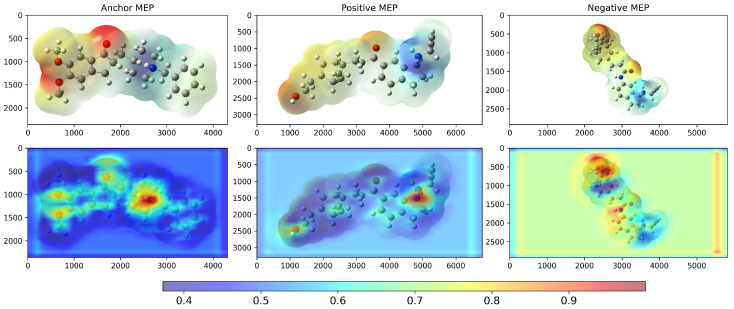
Grad-CAM (**top**) result of the model after training on the potential AChEI dataset, considering the highest and lowest $d^{-1}$ values computed by the model. The X and Y coordinates represent the width and height, respectively, of the original MEP images (**bottom**) in pixel units. The colorbar scale represents the normalized activation intensity (0.0–1.0), where higher values indicate regions that receive more attention from the model during classification. Anchor = DNP, Positive = SCS, Negative = 3AD.

The highest model-predicted similarity to DNP was with SCS ($d^{-1}=0.296$), while the lowest was with 3AD ($d^{-1}=0.154$). For Tanimoto similarity, the highest value in relation to DNP was obtained for 3SS ($S_{T}=0.143$), and the lowest value was observed with SAS ($S_{T}=0.103$). Notwithstanding, the Tanimoto similarity between DNP and SCS was the second lowest ($S_{T}=0.104$), reinforcing the model’s ability to infer similarity based on features present in the input images (as shown in Figure 10), such as spatial positioning, shape, electrostatic potential distribution, and texture, which are associated with types of non-covalent interactions as captured in RDG diagrams and encoded in the GLCM feature vector. It can be observed that the model correctly focused attention on the positions of the bioisosteric centers of DNP (protonated nitrogen, carbonyl, and two ether oxygens O1 and O2) to assign similarity (Figure 10).

**Figure 11:**
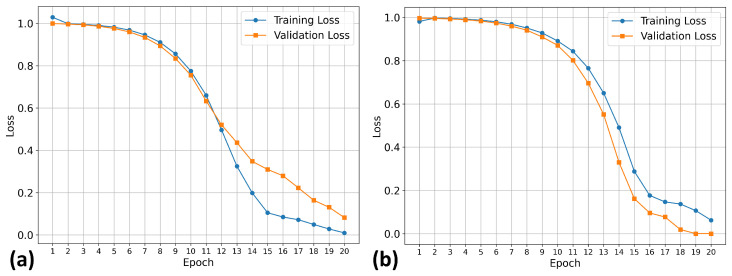
Model’s performance evaluated during training and validation for the potential AChEI dataset: (**a**) model trained with TKI molecules dataset; (**b**) model trained with AChEI dataset.

**Figure 12:**
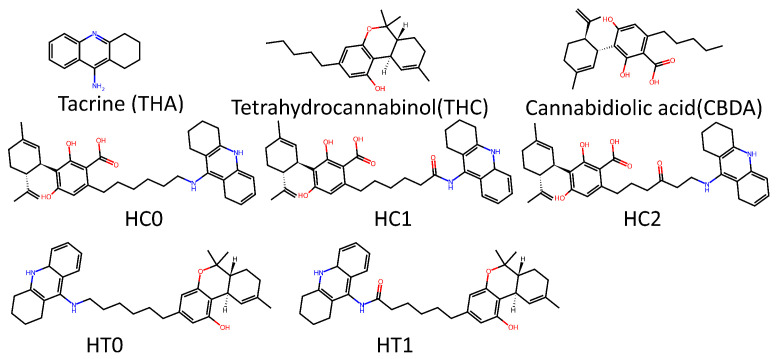
2D structures for major molecules for AChEIs based tacrine-thc-cbda dataset.

In order to evaluate the performance differences between trained models with potential TKI molecules and those trained with potential AChEI enzyme inhibitors (Figure 11), both were tested on a smaller dataset composed of 14 molecules (Figure 12 shows the main ones). These molecules are theoretical heterodimers formed from the structures of THA, THC, and CBDA.

**Figure 13:**
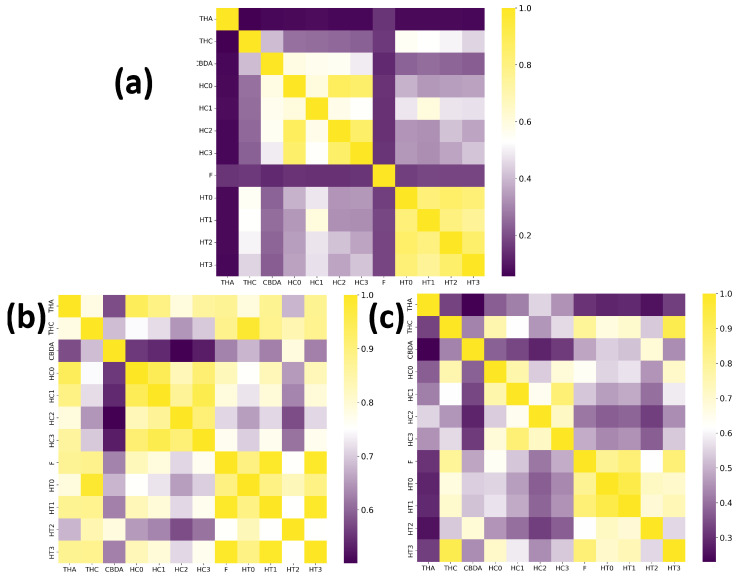
(**a**) Tanimoto similarity based on SMILES representations, compared to model-derived similarity using inverse distance, (**b**) model trained on TKI molecules, (**c**) model trained on potential AChEI inhibitors. Data refer to heterodimers composed of THA, THC, and CBDA.

The comparative analysis of the similarity matrices based on inverse distances, shown in Figure 13, reveals significant differences in the specificity of the models. The model trained with potential AChEI inhibitors, which was exposed to a larger number of *hard triplets* during training, demonstrated a more stringent capacity for molecular discrimination, as suggested by consistently lower similarity values ($d^{-1}$) when compared to the model trained with TKI molecules and also by the model’s performances during training (Figure 11).

For instance, the similarity between THA and THC decreased from $d^{-1}=0.782$ in the TKI molecules model to $d^{-1}=0.333$ in the AChEI model, highlighting the greater specificity of the latter in assessing molecular similarity.

Similarly, the AChEI-trained model exhibited reduced intra-group similarities (e.g., HC0-HC1: $d^{-1}=0.765$ versus $d^{-1}=0.944$ in the TKI molecules model), reinforcing its greater selectivity in similarity identification. Moreover, Figure 13 also shows that both inverse distance models outperformed the Tanimoto similarity coefficients, which are limited to structural comparisons based on the SMILES representation. For example, the Tanimoto score $S_{T}$ between THA and THC was only 0.056, failing to capture the electrostatic and spatial similarities that the inverse distance models successfully identified through MEP maps and RDG-based descriptors. All results are detailed in Tables S5–S7 of the Supplementary Materials.

This difference highlights the importance of incorporating spatial and electronic structure information—such as MEP maps and RDG diagrams—into molecular similarity models. These representations capture non-covalent interactions and their spatial distribution around the molecules, which are essential for a more accurate assessment of similarity.

**Figure 14:**
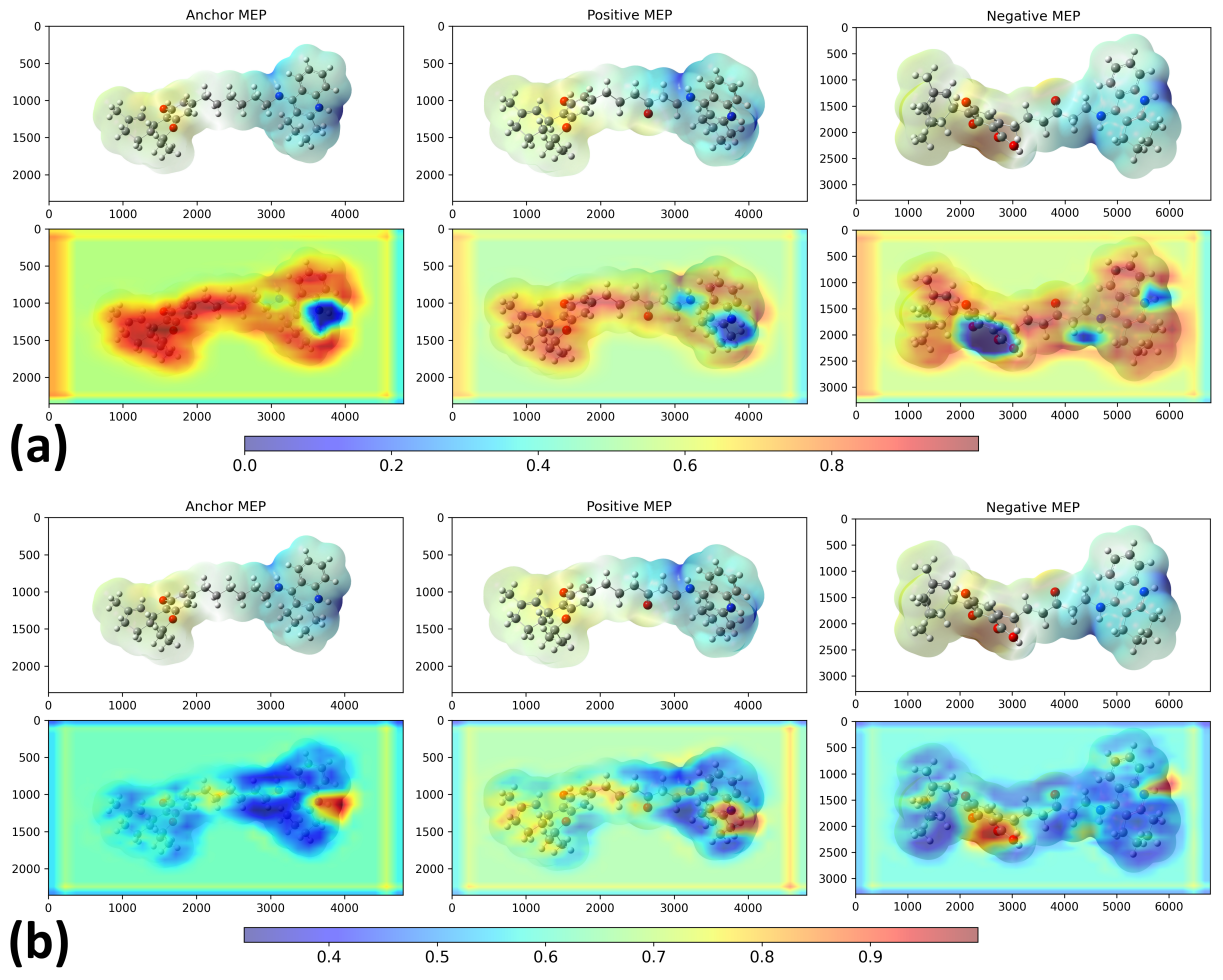
Grad-CAM activation maps generated for the heterodimer similarity prediction task: (**a**) trained with TKIs dataset; (**b**) trained with potential AChEI inhibitors.

**Figure 15:**
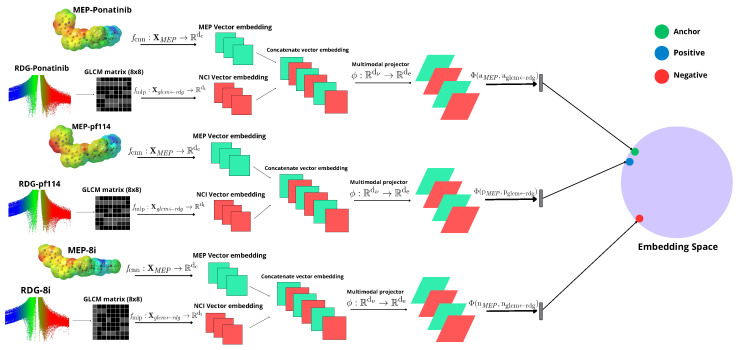
A multimodal architecture processes triplet inputs. An MLP converts RDG diagrams to NCI vector embeddings, while a CNN generates MEP vector embeddings from images. The concatenated vectors are projected into a shared space and evaluated using a triplet loss function. The Supplementary Material shows details of architecture and implementation.

Figure 14 compares Grad-CAM activation maps for the heterodimer similarity task (validation dataset) under two training regimes using the same architecture: Figure 15a trained on potential TKI molecules and Figure 15b trained on potential AChEI inhibitors (huprine and ladostigil-based dataset). In both panels, from left to right, we show HT0 (anchor), HT1 (positive), and HC2 (negative). Together, these panels show the impact of using *hard triplets* and selecting an appropriate training dataset. When comparing the Grad-CAM heatmaps for HT0 (anchor), HT1 (positive), and HC2 (negative), the model trained on potential AChEI inhibitors (b) focused on more specific molecular regions—such as the nitrogen atom (N1) in THA from its pyridine ring, the THC-derived region in HT0, and the oxygen atoms in HC2.

In contrast, the Grad-CAM visualization from the TKI molecules model (a) showed a more diffuse and nonspecific pattern of attention across the molecules. This increased specificity found in the AChEI-based model highlights the relevance of training molecular similarity models with a triplet loss function using *hard triplets*, as well as the need to create a training dataset that is both representative and chemically relevant.

## 3. Methods

### 3.1. Datasets

We utilized three datasets from the literature and generated by our research group. The molecules in this dataset were calculated using density functional theory (DFT) with the Becke, 3-parameter, Lee-Yang-Parr (B3LYP) hybrid functional [27,28,29,30], a widely used exchange-correlation functional that combines Hartree-Fock exchange with DFT correlation. The B3LYP/6-311 + G (d,p) level under the solvation model density (SMD) approach was used [31]. The SMILES strings for all molecules of the dataset are available in the Supplementary Materials. The datasets are also available in the https://github.com/Rafael-Campos-unb/LQC-Bio-dataset repository, acessed on 8 november 2020.

The protonation states of all compounds were optimized for pharmacological applications, prioritizing drug-like properties and aqueous solubility rather than thermodynamic stability alone. The stereochemistry was preserved throughout our computational workflow, with all chiral centers and geometric configurations from the starting ligands that showed the highest enzyme affinity being maintained in the derivative structures.

The first dataset consists of 62 images (31 Molecular Electrostatic Potential, MEP, and 31 Reduced Density Gradient, RDG) of potential TKI molecules. The second dataset includes 62 images (31 MEP and 31 RDG) of potential acetylcholinesterase inhibitors (AChEIs), which are heterodimers based on Huprine W and ladostigil. The third is a cross-validation dataset composed of 24 images (12 MEP and 12 RDG) of heterodimers also proposed by our group as potential AChEIs, based on the molecules tacrine (THA), $\Delta^{9}$-tetrahydrocannabinol (THC), and cannabidiolic acid (CBDA). It is important to make clear that huprine W and ladostigil are not approved drugs. These are investigational compounds rather than approved drugs. The inclusion of these molecules in our study reflects recent research lines of investigation focused on these promising AChE inhibitor scaffolds.

The electronic density matrices used as input for MEP image generation were calculated using Gaussian 16 software [32]. The MEP images were generated using GaussView4 [33] rendering capabilities. The RDG images were also obtained based on DFT data using Multiwfn [34] version 3.8 and Visual Molecular Dynamics (VMD) software version 1.9.3 for rendering the images [35].

### 3.2. Model Architecture

The model consists of a Siamese neural network that takes as input MEP images combined with the GLCM matrix generated from RDG diagrams. We used Tanimoto similarity values ($S_{T}$) to form the triplets (anchor for the reference image, positive for similar images, and negative for dissimilar images), defined as:

(1) $$S_{T}\left(A,B\right)=\frac{A\cap B}{A\cup B},$$

where A and B are two binary molecular fingerprints obtained from the SMILES strings, which are generated using Morgan fingerprints with a fixed size of 2048 bits and a radius of 2. To evaluate the model’s capability to learn distances, we compared the Tanimoto similarity matrix with the inverse distance matrix, where each inverse distance element $d^{-1}$ is calculated using the following expression:

(2) $$d^{-1}=Sim\left(x,y\right)=\frac{1}{1+d(x,y)}$$

where d(x,y) is the distance predicted by the model. The triplets were defined by setting positives as those with $S_{T}\geq 0.70$ relative to the anchor, and negatives as those with $S_{T}\leq 0.40$.

The model architecture (Figure 15) receives an input set for the MEP images and for the GLCM matrices generated from the corresponding RDG diagram images, given that the GLCM matrices were defined with eight levels (8 × 8). The architecture represented in the image has three interconnected components for each element of the triplet: the upper pathway processes MEP images through a convolutional neural network (CNN) to extract spatial-visual features; the lower pathway processes GLCM matrices (8 × 8) derived from RDG diagrams through a multilayer perceptron (MLP) to capture texture information; and the central concatenation mechanism that connects both feature vectors into a unified representation. For example, the anchor (reference image, green) is close to the positive (blue) due to similar images, and far from the negative point (dissimilar images, red).

The cost function that the model aims to minimize is the triplet loss [36], as it is a Siamese network designed to learn similarity and dissimilarity between images. The function operates in a shared embedding space, illustrated as a sphere in the figure, enabling the model to learn meaningful molecular representations by comparing anchor, positive, and negative samples simultaneously.

## 4. Conclusions

The construction of a Siamese neural network model coupled with an MLP for predicting molecular similarity, based on MEP maps and RDG diagrams, was successfully implemented. The results suggest the potential of CNNs in molecular similarity analysis through images, as these networks can capture complex spatial patterns that are relevant for identifying similarities between drug candidates.

The combined use of MEP maps and RDG diagrams has proven promising in the context of drug design, as non-covalent interactions—particularly their spatial arrangement around the molecule—play a central role in defining pharmacological similarity, even among compounds with distinct chemical structures. Additionally, the proposed methodology addresses critical limitations of traditional structural descriptors by incorporating non-covalent interactions and spatial information. For example, when compared to traditional fingerprint methods, the model consistently outperformed Tanimoto similarity calculations based on Morgan fingerprints, particularly in identifying functionally similar compounds with distinct structural scaffolds. For instance, while compound 2k exhibited low Tanimoto similarity to ponatinib ($S_{T}=0.221$), the model correctly identified high electrostatic similarity ($d^{-1}=0.978$), demonstrating the value of incorporating MEP and RDG information.

The model’s performance varied significantly across different biological targets, highlighting the importance of target-specific training. For BCR-ABL inhibitors, Grad-CAM analysis revealed that the model correctly focused on pharmacophoric regions, particularly the piperazine moiety in ponatinib and analogous regions in similar compounds, successfully identifying modifications in purine cores while maintaining recognition of essential binding features.

In the second dataset, the model demonstrated effective performance in identifying potential AChEI candidates, with Grad-CAM visualizations confirming focus on important bioisosteric centers for the AChEIs system, including protonated nitrogen, carbonyl, and ether oxygen atoms. The use of *hard triplets* during the training of this dataset demonstrated enhanced discrimination capabilities compared to the TKIs dataset, with the AChEI-trained model showing more rigorous molecular discrimination as indicated by consistently lower similarity values than the TKI-trained model when tested on the heterodimer validation set (e.g., THA-THC similarity: $d^{-1}=0.333$ vs. $d^{-1}=0.782$, respectively).

Despite these encouraging initial results, additional studies with larger and more diverse datasets are required to validate both models and the proposed methodology more robustly. However, the current lack of large-scale databases containing MEP and RDG images represents a significant limitation for advancing deep learning approaches in this field. Therefore, the scientific community needs to invest in the development, curation, and public availability of such datasets to enable more comprehensive, comparative, and generalizable future research in molecular modeling for drug discovery. 

## Data Availability

The source code of this study is freely available at GitHub, accessed on 8 november 2025 (https://github.com/Rafael-Campos-unb/NCIVISION). The original contributions presented in this study are included in the article/Supplementary Materials. Further inquiries can be directed to the corresponding author.

## Supplementary Materials

The following supporting information can be downloaded at: https://www.mdpi.com/article/10.3390/molecules30234589/s1.
