# Supplementary material for: NCIVISION: A Siamese Neural Network for Molecular Similarity Prediction MEP and RDG Images
Source: Molecules. 2025 Nov 28;30(23):4589. doi: 10.3390/molecules30234589 (PMC12693297; doi:10.3390/molecules30234589)
Supplement: Supplementary file 1 [file molecules-30-04589-s001.zip › molecules-3950284-supplementary.pdf]

NCIVISION: A Siamese Neural Network for Molecular Similarity Prediction MEP and RDG Images

Rafael Campos Vieira,[†] Érica C. M. Nascimento,[‡] Letícia A. Nascimento,[†] Arthur Alves Nascimento,[‡] Nicolas Ricardo de Melo Alves,[‡] and João B. L. Martins[*,†,‡]

†*Department of Pharmacy, Faculty of Health Sciences, University of Brasília, Brasília, Brazil*

‡*Institute of Chemistry, University of Brasília, Brasília, Brazil*

E-mail: lopes@unb.br

Phone: +556131073886

Supporting Information Available

Table S1: Tanimoto similarity values S_T calculated with RDKit for TKI molecules.

MOLECULE	pona	PF-114	7a	7b	7c	7d	7e	7f	8a	8b	8c	8d	8e	8f	8g	8h	8i	8j	8k	8l	2a	2b	2c	2d	2e	2f	2g	2h	2i	2j	2k
pona	1.0	0.679	0.725	0.688	0.659	0.644	0.667	0.651	0.663	0.712	0.766	0.728	0.728	0.694	0.651	0.651	0.636	0.644	0.667	0.651	0.484	0.25	0.255	0.257	0.28	0.34	0.404	0.195	0.198	0.2	0.221
PF-114	0.679	1.0	0.688	0.692	0.643	0.628	0.651	0.635	0.667	0.696	0.684	0.651	0.651	0.621	0.58	0.58	0.567	0.573	0.651	0.635	0.286	0.269	0.274	0.276	0.264	0.17	0.225	0.211	0.214	0.216	0.205
7a	0.725	0.688	1.0	0.843	0.753	0.734	0.763	0.722	0.737	0.747	0.733	0.696	0.696	0.663	0.619	0.619	0.605	0.612	0.718	0.679	0.307	0.264	0.269	0.272	0.31	0.185	0.243	0.205	0.209	0.211	0.245
7b	0.688	0.692	0.843	1.0	0.784	0.763	0.795	0.797	0.767	0.778	0.764	0.724	0.724	0.688	0.642	0.642	0.627	0.634	0.747	0.705	0.277	0.272	0.277	0.28	0.347	0.157	0.215	0.211	0.215	0.217	0.277
7c	0.659	0.643	0.753	0.784	1.0	0.867	0.803	0.738	0.688	0.696	0.727	0.691	0.691	0.659	0.616	0.616	0.602	0.628	0.712	0.738	0.274	0.28	0.286	0.288	0.327	0.159	0.214	0.221	0.225	0.227	0.262
7d	0.644	0.628	0.734	0.763	0.867	1.0	0.782	0.72	0.671	0.679	0.709	0.675	0.675	0.644	0.602	0.602	0.589	0.614	0.695	0.659	0.269	0.275	0.28	0.283	0.32	0.157	0.211	0.217	0.221	0.223	0.257
7e	0.667	0.651	0.763	0.795	0.803	0.782	1.0	0.747	0.696	0.705	0.737	0.766	0.7	0.667	0.683	0.624	0.667	0.675	0.744	0.683	0.276	0.259	0.264	0.267	0.33	0.161	0.216	0.202	0.205	0.207	0.264
7f	0.651	0.635	0.722	0.797	0.738	0.72	0.747	1.0	0.679	0.688	0.718	0.683	0.683	0.651	0.609	0.609	0.596	0.602	0.704	0.667	0.271	0.314	0.333	0.262	0.484	0.158	0.212	0.252	0.269	0.204	0.402
8a	0.663	0.667	0.737	0.767	0.688	0.671	0.696	0.679	1.0	0.747	0.733	0.696	0.696	0.663	0.619	0.619	0.605	0.612	0.696	0.7	0.282	0.276	0.282	0.284	0.297	0.164	0.22	0.216	0.22	0.222	0.234
8b	0.712	0.696	0.747	0.778	0.696	0.679	0.705	0.688	0.747	1.0	0.792	0.75	0.75	0.712	0.667	0.667	0.651	0.659	0.705	0.688	0.297	0.267	0.272	0.275	0.287	0.187	0.234	0.207	0.211	0.213	0.224
8c	0.766	0.684	0.733	0.764	0.727	0.709	0.737	0.718	0.733	0.792	1.0	0.833	0.833	0.789	0.74	0.74	0.722	0.731	0.737	0.718	0.327	0.269	0.275	0.29	0.303	0.2	0.26	0.209	0.213	0.226	0.238
8d	0.728	0.651	0.696	0.724	0.691	0.675	0.766	0.683	0.696	0.75	0.833	1.0	0.863	0.818	0.89	0.769	0.867	0.878	0.744	0.704	0.314	0.259	0.264	0.279	0.291	0.193	0.25	0.202	0.205	0.218	0.229
8e	0.728	0.651	0.696	0.724	0.691	0.675	0.7	0.683	0.696	0.75	0.833	0.863	1.0	0.842	0.769	0.89	0.75	0.759	0.7	0.683	0.314	0.259	0.264	0.279	0.291	0.193	0.25	0.202	0.205	0.218	0.229
8f	0.694	0.621	0.663	0.688	0.659	0.644	0.667	0.651	0.663	0.712	0.789	0.818	0.842	1.0	0.775	0.797	0.714	0.723	0.687	0.651	0.302	0.25	0.255	0.269	0.28	0.186	0.241	0.195	0.198	0.211	0.221
8g	0.651	0.58	0.619	0.642	0.616	0.602	0.683	0.609	0.619	0.667	0.74	0.89	0.769	0.775	1.0	0.867	0.868	0.855	0.663	0.628	0.271	0.221	0.225	0.239	0.25	0.2	0.212	0.168	0.171	0.183	0.193
8h	0.651	0.58	0.619	0.642	0.616	0.602	0.624	0.609	0.619	0.667	0.74	0.769	0.89	0.797	0.867	1.0	0.753	0.741	0.624	0.609	0.271	0.221	0.225	0.239	0.25	0.2	0.212	0.168	0.171	0.183	0.193
8i	0.636	0.567	0.605	0.627	0.602	0.589	0.667	0.596	0.605	0.651	0.722	0.867	0.75	0.714	0.868	0.753	1.0	0.833	0.647	0.614	0.266	0.217	0.221	0.234	0.245	0.186	0.209	0.165	0.168	0.179	0.19
8j	0.644	0.573	0.612	0.634	0.628	0.614	0.675	0.602	0.612	0.659	0.731	0.878	0.759	0.723	0.855	0.741	0.833	1.0	0.655	0.621	0.269	0.219	0.223	0.236	0.248	0.188	0.211	0.167	0.169	0.181	0.191
8k	0.667	0.651	0.718	0.747	0.712	0.695	0.744	0.704	0.696	0.705	0.737	0.744	0.7	0.687	0.663	0.624	0.647	0.655	1.0	0.816	0.276	0.259	0.264	0.267	0.304	0.161	0.216	0.202	0.205	0.207	0.241
8l	0.651	0.635	0.679	0.705	0.738	0.659	0.683	0.667	0.7	0.688	0.718	0.704	0.683	0.651	0.628	0.609	0.614	0.621	0.816	1.0	0.271	0.255	0.259	0.262	0.286	0.158	0.212	0.198	0.202	0.204	0.225
2a	0.484	0.286	0.307	0.277	0.274	0.269	0.276	0.271	0.282	0.297	0.327	0.314	0.314	0.302	0.271	0.271	0.266	0.269	0.276	0.271	1.0	0.489	0.571	0.578	0.617	0.641	0.847	0.406	0.478	0.483	0.517
2b	0.25	0.269	0.264	0.272	0.28	0.275	0.259	0.314	0.276	0.267	0.269	0.259	0.259	0.25	0.221	0.221	0.217	0.219	0.259	0.255	0.489	1.0	0.836	0.705	0.602	0.287	0.406	0.851	0.709	0.595	0.506
2c	0.255	0.274	0.269	0.277	0.286	0.28	0.264	0.333	0.282	0.272	0.275	0.264	0.264	0.255	0.225	0.225	0.221	0.223	0.264	0.259	0.571	0.836	1.0	0.795	0.724	0.333	0.478	0.709	0.847	0.671	0.61
2d	0.257	0.276	0.272	0.28	0.288	0.283	0.267	0.262	0.284	0.275	0.29	0.279	0.279	0.269	0.239	0.239	0.234	0.236	0.267	0.262	0.578	0.705	0.795	1.0	0.605	0.337	0.483	0.595	0.671	0.845	0.506
2e	0.28	0.264	0.31	0.347	0.327	0.32	0.33	0.484	0.297	0.287	0.303	0.291	0.291	0.28	0.25	0.25	0.245	0.248	0.304	0.286	0.617	0.602	0.724	0.605	1.0	0.366	0.517	0.506	0.61	0.506	0.845
2f	0.34	0.17	0.185	0.157	0.159	0.157	0.161	0.158	0.164	0.187	0.2	0.193	0.193	0.186	0.2	0.2	0.186	0.188	0.161	0.158	0.641	0.287	0.333	0.337	0.366	1.0	0.536	0.224	0.265	0.267	0.293
2g	0.404	0.225	0.243	0.215	0.214	0.211	0.216	0.212	0.22	0.234	0.26	0.25	0.25	0.241	0.212	0.212	0.209	0.211	0.216	0.212	0.847	0.406	0.478	0.483	0.517	0.536	1.0	0.495	0.576	0.583	0.622
2h	0.195	0.211	0.205	0.211	0.221	0.217	0.202	0.252	0.216	0.207	0.209	0.202	0.202	0.195	0.168	0.168	0.165	0.167	0.202	0.198	0.406	0.851	0.709	0.595	0.506	0.224	0.495	1.0	0.838	0.709	0.607
2i	0.198	0.214	0.209	0.215	0.225	0.221	0.205	0.269	0.22	0.211	0.213	0.205	0.205	0.198	0.171	0.171	0.168	0.169	0.205	0.202	0.478	0.709	0.847	0.671	0.61	0.265	0.576	0.838	1.0	0.797	0.727
2j	0.200	0.216	0.211	0.217	0.227	0.223	0.207	0.204	0.222	0.213	0.226	0.218	0.218	0.211	0.183	0.183	0.179	0.181	0.207	0.204	0.483	0.595	0.671	0.845	0.506	0.267	0.583	0.709	0.797	1.0	0.61
2k	0.221	0.205	0.245	0.277	0.262	0.257	0.264	0.402	0.234	0.224	0.238	0.229	0.229	0.221	0.193	0.193	0.19	0.191	0.241	0.225	0.517	0.506	0.61	0.506	0.845	0.293	0.622	0.607	0.727	0.61	1.0

Table S2: Inverse distance values d^{-1} calculated by the model for the TKI molecules.

MOLECULE	pona	PF-114	7a	7b	7c	7d	7e	7f	8a	8b	8c	8d	8e	8f	8g	8h	8i	8j	8k	8l	2a	2b	2c	2d	2e	2f	2g	2h	2i	2j	2k
pona	1.0	0.826	0.786	0.748	0.611	0.791	0.547	0.573	0.925	0.79	0.741	0.671	0.725	0.747	0.692	0.631	0.631	0.774	0.682	0.929	0.861	0.727	0.971	0.813	0.828	0.94	0.964	0.907	0.928	0.852	0.978
PF-114	0.826	1.0	0.942	0.888	0.701	0.949	0.618	0.652	0.885	0.947	0.878	0.781	0.856	0.886	0.81	0.728	0.728	0.924	0.796	0.777	0.953	0.858	0.847	0.98	0.992	0.871	0.802	0.902	0.776	0.722	0.812
7a	0.786	0.942	1.0	0.939	0.733	0.991	0.643	0.68	0.839	0.99	0.928	0.821	0.903	0.937	0.852	0.762	0.763	0.979	0.837	0.741	0.9	0.906	0.805	0.959	0.938	0.827	0.764	0.855	0.741	0.691	0.773
7b	0.748	0.888	0.939	1.0	0.769	0.932	0.67	0.711	0.796	0.933	0.988	0.867	0.959	0.996	0.902	0.801	0.802	0.957	0.886	0.707	0.851	0.962	0.765	0.903	0.884	0.785	0.728	0.81	0.707	0.662	0.737
7c	0.611	0.701	0.733	0.769	1.0	0.728	0.839	0.903	0.643	0.729	0.777	0.872	0.795	0.771	0.839	0.95	0.949	0.744	0.854	0.584	0.678	0.793	0.622	0.711	0.699	0.635	0.598	0.652	0.584	0.552	0.603
7d	0.791	0.949	0.991	0.932	0.728	1.0	0.639	0.676	0.845	0.992	0.921	0.815	0.896	0.93	0.846	0.757	0.758	0.972	0.832	0.746	0.907	0.899	0.81	0.967	0.945	0.832	0.769	0.861	0.746	0.695	0.778
7e	0.547	0.618	0.643	0.67	0.839	0.639	1.0	0.922	0.572	0.64	0.676	0.747	0.69	0.671	0.723	0.804	0.803	0.651	0.734	0.525	0.6	0.688	0.556	0.626	0.617	0.566	0.536	0.579	0.525	0.499	0.541
7f	0.573	0.652	0.68	0.711	0.903	0.676	0.922	1.0	0.601	0.677	0.717	0.798	0.733	0.712	0.77	0.863	0.862	0.689	0.783	0.549	0.632	0.731	0.583	0.661	0.651	0.595	0.562	0.609	0.549	0.522	0.567
8a	0.925	0.885	0.839	0.796	0.643	0.845	0.572	0.601	1.0	0.844	0.788	0.709	0.77	0.795	0.733	0.665	0.665	0.825	0.722	0.864	0.925	0.772	0.951	0.87	0.888	0.98	0.895	0.979	0.863	0.797	0.908
8b	0.79	0.947	0.99	0.933	0.729	0.992	0.64	0.677	0.844	1.0	0.923	0.816	0.898	0.932	0.848	0.758	0.759	0.974	0.833	0.745	0.905	0.9	0.809	0.963	0.942	0.831	0.768	0.859	0.744	0.694	0.777
8c	0.741	0.878	0.928	0.988	0.777	0.921	0.676	0.717	0.788	0.923	1.0	0.876	0.971	0.99	0.912	0.809	0.81	0.946	0.895	0.701	0.842	0.973	0.758	0.893	0.875	0.777	0.722	0.802	0.701	0.656	0.73
8d	0.671	0.781	0.821	0.867	0.872	0.815	0.747	0.798	0.709	0.816	0.876	1.0	0.9	0.868	0.957	0.914	0.915	0.835	0.976	0.638	0.752	0.897	0.684	0.793	0.779	0.7	0.655	0.72	0.638	0.601	0.662
8e	0.725	0.856	0.903	0.959	0.795	0.896	0.69	0.733	0.77	0.898	0.971	0.9	1.0	0.961	0.938	0.83	0.83	0.92	0.92	0.687	0.821	0.989	0.741	0.87	0.852	0.759	0.706	0.783	0.687	0.644	0.714
8f	0.747	0.886	0.937	0.996	0.771	0.93	0.671	0.712	0.795	0.932	0.99	0.868	0.961	1.0	0.904	0.803	0.804	0.955	0.887	0.706	0.849	0.963	0.763	0.901	0.882	0.783	0.727	0.808	0.706	0.661	0.735
8g	0.692	0.81	0.852	0.902	0.839	0.846	0.723	0.77	0.733	0.848	0.912	0.957	0.938	0.904	1.0	0.878	0.879	0.867	0.979	0.657	0.779	0.934	0.706	0.823	0.807	0.723	0.675	0.745	0.657	0.617	0.682
8h	0.631	0.728	0.762	0.801	0.95	0.757	0.804	0.863	0.665	0.758	0.809	0.914	0.83	0.803	0.878	1.0	0.998	0.774	0.894	0.602	0.703	0.827	0.643	0.738	0.726	0.657	0.617	0.675	0.602	0.569	0.623
8i	0.631	0.728	0.763	0.802	0.949	0.758	0.803	0.862	0.665	0.759	0.81	0.915	0.83	0.804	0.879	0.998	1.0	0.775	0.895	0.602	0.703	0.828	0.643	0.739	0.726	0.658	0.617	0.675	0.602	0.569	0.623
8j	0.774	0.924	0.979	0.957	0.744	0.972	0.651	0.689	0.825	0.974	0.946	0.835	0.92	0.955	0.867	0.774	0.775	1.0	0.852	0.73	0.884	0.922	0.792	0.94	0.92	0.813	0.752	0.84	0.73	0.682	0.761
8k	0.682	0.796	0.837	0.886	0.854	0.832	0.734	0.783	0.722	0.833	0.895	0.976	0.92	0.887	0.979	0.894	0.895	0.852	1.0	0.648	0.766	0.917	0.696	0.809	0.794	0.712	0.666	0.733	0.648	0.61	0.673
8l	0.929	0.777	0.741	0.707	0.584	0.746	0.525	0.549	0.864	0.745	0.701	0.638	0.687	0.706	0.657	0.602	0.602	0.73	0.648	1.0	0.807	0.688	0.904	0.765	0.779	0.877	0.96	0.848	0.993	0.911	0.946
2a	0.861	0.953	0.9	0.851	0.678	0.907	0.6	0.632	0.925	0.905	0.842	0.752	0.821	0.849	0.779	0.703	0.703	0.884	0.766	0.807	1.0	0.824	0.883	0.936	0.957	0.91	0.835	0.944	0.807	0.749	0.846
2b	0.727	0.858	0.906	0.962	0.793	0.899	0.688	0.731	0.772	0.9	0.973	0.897	0.989	0.963	0.934	0.827	0.828	0.922	0.917	0.688	0.824	1.0	0.743	0.873	0.855	0.762	0.708	0.785	0.688	0.645	0.716
2c	0.971	0.847	0.805	0.765	0.622	0.81	0.556	0.583	0.951	0.809	0.758	0.684	0.741	0.763	0.706	0.643	0.643	0.792	0.696	0.904	0.883	0.743	1.0	0.833	0.849	0.967	0.938	0.932	0.904	0.831	0.952
2d	0.813	0.98	0.959	0.903	0.711	0.967	0.626	0.661	0.87	0.963	0.893	0.793	0.87	0.901	0.823	0.738	0.739	0.94	0.809	0.765	0.936	0.873	0.833	1.0	0.977	0.856	0.79	0.886	0.765	0.712	0.799
2e	0.828	0.992	0.938	0.884	0.699	0.945	0.617	0.651	0.888	0.942	0.875	0.779	0.852	0.882	0.807	0.726	0.726	0.920	0.794	0.779	0.957	0.855	0.849	0.977	1.0	0.874	0.805	0.905	0.779	0.724	0.815
2f	0.94	0.871	0.827	0.785	0.635	0.832	0.566	0.595	0.98	0.831	0.777	0.7	0.759	0.783	0.723	0.657	0.658	0.813	0.712	0.877	0.91	0.762	0.967	0.856	0.874	1.0	0.91	0.961	0.877	0.808	0.923
2g	0.964	0.802	0.764	0.728	0.598	0.769	0.536	0.562	0.895	0.768	0.722	0.655	0.706	0.727	0.675	0.617	0.617	0.752	0.666	0.96	0.835	0.708	0.938	0.79	0.805	0.91	1.0	0.878	0.961	0.879	0.984
2h	0.907	0.902	0.855	0.81	0.652	0.861	0.579	0.609	0.979	0.859	0.802	0.72	0.783	0.808	0.745	0.675	0.675	0.84	0.733	0.848	0.944	0.785	0.932	0.886	0.905	0.961	0.878	1.0	0.848	0.783	0.89
2i	0.928	0.776	0.741	0.707	0.584	0.746	0.525	0.549	0.863	0.744	0.701	0.638	0.687	0.706	0.657	0.602	0.602	0.73	0.648	0.993	0.807	0.688	0.904	0.765	0.779	0.877	0.961	0.848	1.0	0.911	0.946
2j	0.852	0.722	0.691	0.662	0.552	0.695	0.499	0.522	0.797	0.694	0.656	0.601	0.644	0.661	0.617	0.569	0.569	0.682	0.61	0.911	0.749	0.645	0.831	0.712	0.724	0.808	0.879	0.783	0.911	1.0	0.867
2k	0.978	0.812	0.773	0.737	0.603	0.778	0.541	0.567	0.908	0.777	0.73	0.662	0.714	0.735	0.682	0.623	0.623	0.761	0.673	0.946	0.846	0.716	0.952	0.799	0.815	0.923	0.984	0.89	0.946	0.867	1.0

Table S3: Tanimoto similarity values S_T calculated with RDKit for candidate AChEI molecules.

MOLECULE	DNP	3AD	3AS	3CD	3CS	3ED	3ES	3HD	3HS	3SD	3SS	5AD	5AS	5CD	5CS	5ED	5ES	5HD	5HS	5SD	5SS	SAD	SAS	SCD	SCS	SED	SES	SHD	SHS	SSD	SSS
DNP	1.0	0.117	0.131	0.121	0.135	0.121	0.135	0.125	0.14	0.129	0.143	0.113	0.127	0.117	0.131	0.118	0.133	0.124	0.137	0.125	0.139	0.108	0.101	0.111	0.104	0.12	0.104	0.115	0.108	0.111	0.112
3AD	0.117	1.0	0.757	0.738	0.579	0.638	0.519	0.667	0.539	0.706	0.557	0.889	0.726	0.681	0.557	0.62	0.506	0.657	0.526	0.653	0.537	0.727	0.644	0.521	0.481	0.5	0.427	0.479	0.443	0.461	0.463
3AS	0.131	0.757	1.0	0.579	0.764	0.519	0.671	0.539	0.699	0.557	0.733	0.726	0.9	0.557	0.711	0.506	0.654	0.532	0.68	0.537	0.684	0.635	0.628	0.475	0.459	0.458	0.409	0.438	0.424	0.422	0.443
3CD	0.121	0.738	0.579	1.0	0.746	0.667	0.539	0.698	0.562	0.738	0.579	0.681	0.557	0.883	0.714	0.647	0.526	0.688	0.547	0.681	0.557	0.521	0.481	0.714	0.629	0.521	0.443	0.5	0.461	0.479	0.481
3CS	0.135	0.579	0.764	0.746	1.0	0.539	0.699	0.562	0.729	0.579	0.764	0.557	0.711	0.714	0.896	0.526	0.68	0.554	0.708	0.557	0.711	0.475	0.459	0.62	0.613	0.475	0.424	0.455	0.439	0.438	0.459
3ED	0.121	0.638	0.519	0.667	0.539	1.0	0.746	0.754	0.583	0.738	0.6	0.657	0.519	0.687	0.538	0.931	0.725	0.742	0.568	0.758	0.597	0.461	0.427	0.479	0.443	0.542	0.629	0.522	0.48	0.714	0.5
3ES	0.135	0.519	0.671	0.539	0.699	0.746	1.0	0.583	0.779	0.6	0.764	0.519	0.688	0.538	0.716	0.725	0.938	0.575	0.757	0.597	0.781	0.422	0.409	0.438	0.424	0.494	0.613	0.474	0.457	0.62	0.476
3HD	0.125	0.667	0.539	0.698	0.562	0.754	0.583	1.0	0.734	0.692	0.539	0.687	0.538	0.719	0.56	0.73	0.568	0.944	0.712	0.687	0.538	0.479	0.443	0.5	0.461	0.5	0.48	0.7	0.612	0.522	0.443
3HS	0.14	0.539	0.699	0.562	0.729	0.583	0.779	0.734	1.0	0.56	0.699	0.538	0.716	0.56	0.746	0.568	0.757	0.723	0.935	0.538	0.716	0.438	0.424	0.455	0.439	0.456	0.457	0.603	0.597	0.474	0.424
3SD	0.129	0.706	0.557	0.738	0.579	0.738	0.6	0.692	0.56	1.0	0.757	0.653	0.537	0.681	0.557	0.716	0.584	0.682	0.545	0.889	0.726	0.5	0.463	0.521	0.481	0.727	0.5	0.5	0.462	0.542	0.644
3SS	0.143	0.557	0.733	0.579	0.764	0.6	0.764	0.539	0.699	0.757	1.0	0.537	0.684	0.557	0.711	0.584	0.743	0.532	0.68	0.726	0.9	0.458	0.443	0.475	0.459	0.635	0.476	0.438	0.424	0.494	0.628
5AD	0.113	0.889	0.726	0.681	0.557	0.657	0.519	0.687	0.538	0.653	0.537	1.0	0.767	0.75	0.575	0.662	0.506	0.701	0.544	0.718	0.554	0.671	0.618	0.5	0.463	0.481	0.429	0.48	0.444	0.462	0.447
5AS	0.127	0.726	0.9	0.557	0.711	0.519	0.688	0.538	0.716	0.537	0.684	0.767	1.0	0.575	0.773	0.506	0.692	0.532	0.72	0.554	0.744	0.61	0.585	0.458	0.443	0.442	0.411	0.439	0.425	0.424	0.429
5CD	0.117	0.681	0.557	0.883	0.714	0.687	0.538	0.719	0.56	0.681	0.557	0.75	0.575	1.0	0.757	0.691	0.525	0.734	0.566	0.75	0.575	0.5	0.463	0.657	0.603	0.5	0.444	0.5	0.462	0.48	0.463
5CS	0.131	0.557	0.711	0.714	0.896	0.538	0.716	0.56	0.746	0.557	0.711	0.575	0.773	0.757	1.0	0.525	0.72	0.553	0.75	0.575	0.773	0.458	0.443	0.595	0.57	0.458	0.425	0.456	0.44	0.439	0.443
5ED	0.118	0.62	0.506	0.647	0.526	0.931	0.725	0.73	0.568	0.716	0.584	0.662	0.506	0.691	0.525	1.0	0.754	0.774	0.595	0.761	0.582	0.449	0.417	0.467	0.432	0.527	0.611	0.507	0.468	0.667	0.488
5ES	0.133	0.506	0.654	0.526	0.68	0.725	0.938	0.568	0.757	0.584	0.743	0.506	0.692	0.525	0.72	0.754	1.0	0.581	0.786	0.582	0.784	0.412	0.4	0.427	0.414	0.481	0.577	0.462	0.446	0.603	0.465
5HD	0.124	0.657	0.532	0.688	0.554	0.742	0.575	0.944	0.723	0.682	0.532	0.701	0.532	0.734	0.553	0.774	0.581	1.0	0.754	0.701	0.532	0.473	0.438	0.493	0.455	0.493	0.474	0.661	0.603	0.514	0.438
5HS	0.137	0.526	0.68	0.547	0.708	0.568	0.757	0.712	0.935	0.545	0.68	0.544	0.72	0.566	0.75	0.595	0.786	0.754	1.0	0.544	0.72	0.427	0.414	0.443	0.429	0.444	0.446	0.586	0.56	0.462	0.414
5SD	0.125	0.653	0.537	0.681	0.557	0.758	0.597	0.687	0.538	0.889	0.726	0.718	0.554	0.75	0.575	0.761	0.582	0.701	0.544	1.0	0.767	0.481	0.447	0.5	0.463	0.671	0.5	0.48	0.444	0.541	0.618
5SS	0.139	0.537	0.684	0.557	0.711	0.597	0.781	0.538	0.716	0.726	0.9	0.554	0.744	0.575	0.773	0.582	0.784	0.532	0.72	0.767	1.0	0.442	0.429	0.458	0.443	0.61	0.477	0.439	0.425	0.494	0.585
SAD	0.108	0.727	0.635	0.521	0.475	0.461	0.422	0.479	0.438	0.5	0.458	0.671	0.61	0.5	0.458	0.449	0.412	0.473	0.427	0.481	0.442	1.0	0.71	0.73	0.533	0.697	0.474	0.656	0.493	0.627	0.513
SAS	0.101	0.644	0.628	0.481	0.459	0.427	0.409	0.443	0.424	0.463	0.443	0.618	0.585	0.463	0.443	0.417	0.4	0.438	0.414	0.447	0.429	0.71	1.0	0.533	0.754	0.513	0.658	0.493	0.686	0.474	0.722
SCD	0.111	0.521	0.475	0.714	0.62	0.479	0.438	0.5	0.455	0.521	0.475	0.5	0.458	0.657	0.595	0.467	0.427	0.493	0.443	0.5	0.458	0.73	0.533	1.0	0.697	0.73	0.493	0.689	0.514	0.656	0.533
SCS	0.104	0.481	0.459	0.629	0.613	0.443	0.424	0.461	0.439	0.481	0.459	0.463	0.443	0.603	0.57	0.432	0.414	0.455	0.429	0.463	0.443	0.533	0.754	0.697	1.0	0.533	0.686	0.514	0.716	0.493	0.754
SED	0.12	0.5	0.458	0.521	0.475	0.542	0.494	0.5	0.456	0.727	0.635	0.481	0.442	0.5	0.458	0.527	0.481	0.493	0.444	0.671	0.61	0.697	0.513	0.73	0.533	1.0	0.554	0.683	0.514	0.73	0.71
SES	0.104	0.427	0.409	0.443	0.424	0.629	0.613	0.48	0.457	0.5	0.476	0.429	0.411	0.444	0.425	0.611	0.577	0.474	0.446	0.5	0.477	0.474	0.658	0.493	0.686	0.554	1.0	0.535	0.769	0.697	0.754
SHD	0.115	0.479	0.438	0.5	0.455	0.522	0.474	0.7	0.603	0.5	0.438	0.48	0.439	0.5	0.456	0.507	0.462	0.661	0.586	0.48	0.439	0.656	0.493	0.689	0.514	0.683	0.535	1.0	0.683	0.746	0.493
SHS	0.108	0.443	0.424	0.461	0.439	0.48	0.457	0.612	0.597	0.462	0.424	0.444	0.425	0.462	0.44	0.468	0.446	0.603	0.56	0.444	0.425	0.493	0.686	0.514	0.716	0.514	0.769	0.683	1.0	0.535	0.686
SSD	0.111	0.461	0.422	0.479	0.438	0.714	0.62	0.522	0.474	0.542	0.494	0.462	0.424	0.48	0.439	0.667	0.603	0.514	0.462	0.541	0.494	0.627	0.474	0.656	0.493	0.73	0.697	0.746	0.535	1.0	0.554
SSS	0.112	0.463	0.443	0.481	0.459	0.5	0.476	0.443	0.424	0.644	0.628	0.447	0.429	0.463	0.443	0.488	0.465	0.438	0.414	0.618	0.585	0.513	0.722	0.533	0.754	0.71	0.754	0.493	0.686	0.554	1.0

Table S4: Inverse distance values d^{-1} calculated by the model for candidate AChEI molecules.

MOLECULE	DNP	3AD	3AS	3CD	3CS	3ED	3ES	3HD	3HS	3SD	3SS	5AD	5AS	5CD	5CS
DNP	1.0	0.154	0.19	0.179	0.222	0.197	0.258	0.215	0.185	0.177	0.196	0.193	0.182	0.181	0.169
3AD	0.154	1.0	0.447	0.522	0.336	0.417	0.277	0.352	0.48	0.541	0.42	0.432	0.505	0.507	0.643
3AS	0.19	0.447	1.0	0.758	0.574	0.858	0.42	0.622	0.867	0.72	0.871	0.922	0.797	0.792	0.595
3CD	0.179	0.522	0.758	1.0	0.485	0.674	0.371	0.519	0.857	0.936	0.681	0.714	0.937	0.944	0.735
3CS	0.222	0.336	0.574	0.485	1.0	0.634	0.611	0.882	0.528	0.47	0.628	0.602	0.501	0.499	0.413
3ED	0.197	0.417	0.858	0.674	0.634	1.0	0.452	0.693	0.759	0.644	0.982	0.923	0.705	0.701	0.542
3ES	0.258	0.277	0.42	0.371	0.611	0.452	1.0	0.565	0.395	0.361	0.448	0.435	0.38	0.379	0.327
3HD	0.215	0.352	0.622	0.519	0.882	0.693	0.565	1.0	0.568	0.501	0.685	0.655	0.537	0.535	0.437
3HS	0.185	0.48	0.867	0.857	0.528	0.759	0.395	0.568	1.0	0.809	0.768	0.81	0.908	0.9	0.655
3SD	0.177	0.541	0.72	0.936	0.47	0.644	0.361	0.501	0.809	1.0	0.651	0.681	0.881	0.887	0.774
3SS	0.196	0.42	0.871	0.681	0.628	0.982	0.448	0.685	0.768	0.651	1.0	0.936	0.713	0.71	0.547
5AD	0.193	0.432	0.922	0.714	0.602	0.923	0.435	0.655	0.81	0.681	0.936	1.0	0.749	0.745	0.568
5AS	0.182	0.505	0.797	0.937	0.501	0.705	0.38	0.537	0.908	0.881	0.713	0.749	1.0	0.978	0.701
5CD	0.181	0.507	0.792	0.944	0.499	0.701	0.379	0.535	0.9	0.887	0.71	0.745	0.978	1.0	0.705
5CS	0.169	0.643	0.595	0.735	0.413	0.542	0.327	0.437	0.655	0.774	0.547	0.568	0.701	0.705	1.0
5ED	0.186	0.474	0.888	0.837	0.536	0.776	0.4	0.577	0.967	0.791	0.786	0.83	0.884	0.88	0.643
5ES	0.178	0.534	0.734	0.958	0.476	0.655	0.365	0.508	0.827	0.97	0.662	0.693	0.903	0.907	0.758
5HD	0.194	0.427	0.901	0.7	0.612	0.947	0.44	0.667	0.792	0.668	0.961	0.973	0.734	0.73	0.559
5HS	0.221	0.338	0.581	0.49	0.98	0.642	0.604	0.897	0.533	0.474	0.635	0.609	0.506	0.504	0.416
5SD	0.186	0.474	0.885	0.838	0.535	0.774	0.399	0.576	0.965	0.793	0.784	0.828	0.885	0.882	0.644
5SS	0.182	0.505	0.798	0.937	0.501	0.705	0.38	0.537	0.909	0.881	0.714	0.75	0.99	0.985	0.701
SAD	0.195	0.424	0.889	0.693	0.618	0.96	0.443	0.673	0.783	0.662	0.974	0.96	0.726	0.723	0.555
SAS	0.21	0.368	0.674	0.555	0.795	0.758	0.528	0.887	0.611	0.534	0.749	0.713	0.575	0.573	0.462
SCD	0.247	0.291	0.455	0.397	0.686	0.492	0.847	0.628	0.425	0.387	0.488	0.472	0.408	0.407	0.347
SCS	0.296	0.243	0.347	0.313	0.468	0.368	0.666	0.44	0.33	0.306	0.366	0.357	0.319	0.318	0.281
SED	0.187	0.466	0.919	0.811	0.547	0.799	0.406	0.59	0.937	0.768	0.81	0.856	0.857	0.851	0.628
SES	0.227	0.324	0.539	0.46	0.898	0.592	0.656	0.802	0.498	0.446	0.586	0.564	0.474	0.473	0.395
SHD	0.185	0.479	0.872	0.852	0.53	0.763	0.396	0.57	0.991	0.805	0.772	0.814	0.903	0.896	0.652
SHS	0.2	0.403	0.801	0.638	0.67	0.921	0.469	0.736	0.713	0.611	0.908	0.856	0.665	0.662	0.518
SSD	0.231	0.316	0.518	0.445	0.841	0.566	0.69	0.755	0.48	0.431	0.561	0.541	0.458	0.456	0.383
SSS	0.21	0.367	0.67	0.552	0.8	0.754	0.53	0.895	0.608	0.532	0.744	0.709	0.573	0.571	0.461

MOLECULE	5ED	5ES	5HD	5HS	5SD	5SS	SAD	SAS	SCD	SCS	SED	SES	SHD	SHS	SSD	SSS
DNP	0.186	0.178	0.194	0.221	0.186	0.182	0.195	0.21	0.247	0.296	0.187	0.227	0.185	0.2	0.231	0.21
3AD	0.474	0.534	0.427	0.338	0.474	0.505	0.424	0.368	0.291	0.243	0.466	0.324	0.479	0.403	0.316	0.367
3AS	0.888	0.734	0.901	0.581	0.885	0.798	0.889	0.674	0.455	0.347	0.919	0.539	0.872	0.801	0.518	0.67
3CD	0.837	0.958	0.7	0.49	0.838	0.937	0.693	0.555	0.397	0.313	0.811	0.46	0.852	0.638	0.445	0.552
3CS	0.536	0.476	0.612	0.98	0.535	0.501	0.618	0.795	0.686	0.468	0.547	0.898	0.53	0.67	0.841	0.8
3ED	0.776	0.655	0.947	0.642	0.774	0.705	0.96	0.758	0.492	0.368	0.799	0.592	0.763	0.921	0.566	0.754
3ES	0.4	0.365	0.44	0.604	0.399	0.38	0.443	0.528	0.847	0.666	0.406	0.656	0.396	0.469	0.69	0.53
3HD	0.577	0.508	0.667	0.897	0.576	0.537	0.673	0.887	0.628	0.44	0.59	0.802	0.57	0.736	0.755	0.895
3HS	0.967	0.827	0.792	0.533	0.965	0.909	0.783	0.611	0.425	0.33	0.937	0.498	0.991	0.713	0.48	0.608
3SD	0.791	0.97	0.668	0.474	0.793	0.881	0.662	0.534	0.387	0.306	0.768	0.446	0.805	0.611	0.431	0.532
3SS	0.786	0.662	0.961	0.635	0.784	0.714	0.974	0.749	0.488	0.366	0.81	0.586	0.772	0.908	0.561	0.744
5AD	0.83	0.693	0.973	0.609	0.828	0.75	0.96	0.713	0.472	0.357	0.856	0.564	0.814	0.856	0.541	0.709
5AS	0.884	0.903	0.734	0.506	0.885	0.99	0.726	0.575	0.408	0.319	0.857	0.474	0.903	0.665	0.458	0.573
5CD	0.88	0.907	0.73	0.504	0.882	0.985	0.723	0.573	0.407	0.318	0.851	0.473	0.896	0.662	0.456	0.571
5CS	0.643	0.758	0.559	0.416	0.644	0.701	0.555	0.462	0.347	0.281	0.628	0.395	0.652	0.518	0.383	0.461
5ED	1.0	0.808	0.811	0.541	0.994	0.886	0.802	0.622	0.43	0.333	0.963	0.505	0.974	0.728	0.487	0.619
5ES	0.808	1.0	0.68	0.48	0.809	0.902	0.674	0.542	0.391	0.309	0.785	0.451	0.823	0.621	0.436	0.54
5HD	0.811	0.68	1.0	0.62	0.809	0.735	0.986	0.727	0.478	0.361	0.836	0.573	0.797	0.876	0.549	0.723
5HS	0.541	0.48	0.62	1.0	0.541	0.506	0.625	0.807	0.677	0.463	0.553	0.883	0.535	0.679	0.827	0.812
5SD	0.994	0.809	0.809	0.541	1.0	0.888	0.8	0.621	0.43	0.333	0.959	0.505	0.972	0.727	0.486	0.618
5SS	0.886	0.902	0.735	0.506	0.888	1.0	0.727	0.576	0.408	0.319	0.858	0.474	0.903	0.666	0.458	0.573
SAD	0.802	0.674	0.986	0.625	0.8	0.727	1.0	0.735	0.482	0.363	0.826	0.577	0.788	0.888	0.553	0.731
SAS	0.622	0.542	0.727	0.807	0.621	0.576	0.735	1.0	0.583	0.417	0.637	0.729	0.613	0.809	0.691	0.985
SCD	0.43	0.391	0.478	0.677	0.43	0.408	0.482	0.583	1.0	0.595	0.438	0.744	0.426	0.513	0.788	0.586
SCS	0.333	0.309	0.361	0.463	0.333	0.319	0.363	0.417	0.595	1.0	0.337	0.494	0.33	0.38	0.513	0.419
SED	0.963	0.785	0.836	0.553	0.959	0.858	0.826	0.637	0.438	0.337	1.0	0.515	0.944	0.749	0.496	0.634
SES	0.505	0.451	0.573	0.883	0.505	0.474	0.577	0.729	0.744	0.494	0.515	1.0	0.5	0.623	0.928	0.733
SHD	0.974	0.823	0.797	0.535	0.972	0.903	0.788	0.613	0.426	0.33	0.944	0.5	1.0	0.717	0.482	0.611
SHS	0.728	0.621	0.876	0.679	0.727	0.666	0.888	0.809	0.513	0.38	0.749	0.623	0.717	1.0	0.595	0.805
SSD	0.487	0.436	0.549	0.827	0.486	0.458	0.553	0.691	0.788	0.513	0.496	0.928	0.482	0.595	1.0	0.695
SSS	0.619	0.54	0.723	0.812	0.618	0.573	0.731	0.985	0.586	0.419	0.634	0.733	0.611	0.805	0.695	1.0

Table S5: Tanimoto similarity values S_T calculated with RDKit for heterodimers design with THA, THC and CBDA.

MOLECULE	THA	THC	CBDA	HC0	HC1	HC2	HC3	F	HT0	HT1	HT2	HT3
THA	1.0	0.056	0.068	0.071	0.08	0.07	0.069	0.155	0.074	0.071	0.071	0.07
THC	0.056	1.0	0.406	0.265	0.257	0.248	0.233	0.16	0.551	0.531	0.506	0.448
CBDA	0.068	0.406	1.0	0.588	0.566	0.554	0.494	0.133	0.24	0.257	0.243	0.229
HC0	0.071	0.265	0.588	1.0	0.602	0.88	0.847	0.15	0.385	0.339	0.348	0.357
HC1	0.08	0.257	0.566	0.602	1.0	0.574	0.538	0.146	0.481	0.602	0.477	0.472
HC2	0.07	0.248	0.554	0.88	0.574	1.0	0.849	0.147	0.33	0.322	0.414	0.362
HC3	0.069	0.233	0.494	0.847	0.538	0.849	1.0	0.155	0.339	0.319	0.362	0.42
F	0.155	0.16	0.133	0.15	0.146	0.147	0.155	1.0	0.172	0.187	0.185	0.184
HT0	0.074	0.551	0.24	0.385	0.481	0.33	0.339	0.172	1.0	0.819	0.854	0.821
HT1	0.071	0.531	0.257	0.339	0.602	0.322	0.319	0.187	0.819	1.0	0.802	0.753
HT2	0.071	0.506	0.243	0.348	0.477	0.414	0.362	0.185	0.854	0.802	1.0	0.847
HT3	0.07	0.448	0.229	0.357	0.472	0.362	0.42	0.184	0.821	0.753	0.847	1.0

Table S6: Inverse distance values (d^{-1}) computed by the model trained on the TKI molecules dataset, applied to the heterodimer cross-validation set.

MOLECULE	THA	THC	CBDA	HC0	HC1	HC2	HC3	F	HT0	HT1	HT2	HT3
THA	1.0	0.782	0.577	0.938	0.89	0.791	0.862	0.872	0.791	0.876	0.68	0.876
THC	0.782	1.0	0.687	0.746	0.716	0.648	0.696	0.882	0.985	0.878	0.84	0.878
CBDA	0.577	0.687	1.0	0.557	0.541	0.501	0.529	0.629	0.68	0.627	0.789	0.627
HC0	0.938	0.746	0.557	1.0	0.944	0.832	0.912	0.828	0.754	0.832	0.653	0.832
HC1	0.89	0.716	0.541	0.944	1.0	0.87	0.952	0.791	0.724	0.795	0.63	0.795
HC2	0.791	0.648	0.501	0.832	0.87	1.0	0.905	0.71	0.655	0.712	0.577	0.712
HC3	0.862	0.696	0.529	0.912	0.952	0.905	1.0	0.767	0.703	0.77	0.614	0.77
F	0.872	0.882	0.629	0.828	0.791	0.71	0.767	1.0	0.894	0.994	0.755	0.993
HT0	0.791	0.985	0.68	0.754	0.724	0.655	0.703	0.894	1.0	0.89	0.829	0.89
HT1	0.876	0.878	0.627	0.832	0.795	0.712	0.77	0.994	0.89	1.0	0.752	0.994
HT2	0.68	0.84	0.789	0.653	0.63	0.577	0.614	0.755	0.829	0.752	1.0	0.752
HT3	0.876	0.878	0.627	0.832	0.795	0.712	0.77	0.993	0.89	0.994	0.752	1.0

Table S7: Inverse distance values (d^{-1}) computed by the model trained on the AChEI inhibitor dataset, applied to the heterodimer cross-validation set

MOLECULE	THA	THC	CBDA	HC0	HC1	HC2	HC3	F	HT0	HT1	HT2	HT3
THA	1.0	0.333	0.231	0.374	0.422	0.55	0.453	0.308	0.286	0.292	0.258	0.325
THC	0.333	1.0	0.428	0.755	0.613	0.459	0.559	0.798	0.67	0.702	0.532	0.924
CBDA	0.231	0.428	1.0	0.376	0.337	0.285	0.32	0.481	0.543	0.523	0.687	0.444
HC0	0.374	0.755	0.376	1.0	0.765	0.539	0.683	0.634	0.55	0.572	0.454	0.711
HC1	0.422	0.613	0.337	0.765	1.0	0.645	0.86	0.531	0.471	0.487	0.399	0.584
HC2	0.55	0.459	0.285	0.539	0.645	1.0	0.719	0.411	0.374	0.384	0.327	0.442
HC3	0.453	0.559	0.32	0.683	0.86	0.719	1.0	0.49	0.438	0.452	0.375	0.534
F	0.308	0.798	0.481	0.634	0.531	0.411	0.49	1.0	0.806	0.854	0.615	0.854
HT0	0.286	0.67	0.543	0.55	0.471	0.374	0.438	0.806	1.0	0.935	0.722	0.708
HT1	0.292	0.702	0.523	0.572	0.487	0.384	0.452	0.854	0.935	1.0	0.688	0.745
HT2	0.258	0.532	0.687	0.454	0.399	0.327	0.375	0.615	0.722	0.688	1.0	0.557
HT3	0.325	0.924	0.444	0.711	0.584	0.442	0.534	0.854	0.708	0.745	0.557	1.0

Table S8: SMILES for TKIs data.

MOLECULE	SMILES
pona	O=C(NC1=CC=C(C(=C1)C(F)(F)F)CN2CCN(C)CC2)C3=CC=C(C(C#CC4=CN=C5C=CC=NN45)=C3)C
pf114	O=C(NC1=CC=C(C(=C1)C(F)(F)F)CN2CCN(C)CC2)C3=CC=C(C(C#CC4=NN=C5C=CC=CN45)=C3)C
7a	O=C(NC1=CC=C(C(=C1)C(F)(F)F)CN2CCN(C)CC2)C3=CC=C(C(C#CC=4C=NC=CC4)=C3)C
7b	O=C(NC1=CC=C(C(=C1)C(F)(F)F)CN2CCN(C)CC2)C3=CC=C(C(C#CC=4C=NC=NC4)=C3)C
7c	O=C(NC1=CC=C(C(=C1)C(F)(F)F)CN2CCN(C)CC2)C3=CC=C(C(C#CC4=CN=C(C=C4)NC(=O)C)=C3)C
7d	O=C(NC1=CC=C(C(=C1)C(F)(F)F)CN2CCN(C)CC2)C3=CC=C(C(C#CC4=CN=C(C=C4)NC(=O)CC)=C3)C
7e	O=C(N)C1=NC=C(C#CC2=CC(=CC=C2C)C(=O)NC3=CC=C(C(=C3)C(F)(F)F)CN4CCN(C)CC4)C=C1
7f	O=C(NC1=CC=C(C(=C1)C(F)(F)F)CN2CCN(C)CC2)C3=CC=C(C(C#CC4=CN=C(N=C4)NC5CC5)=C3)C
8a	O=C(NC1=CC=C(C(=C1)C(F)(F)F)CN2CCN(C)CC2)C3=CC=C(C(C#CC4=CN=CN4)=C3)C
8b	O=C(NC1=CC=C(C(=C1)C(F)(F)F)CN2CCN(C)CC2)C3=CC=C(C(C#CC4=CN=CN4C)=C3)C
8c	O=C(NC1=CC=C(C(=C1)C(F)(F)F)CN2CCN(C)CC2)C3=CC=C(C(C#CC4=CN=C(N4C)C)=C3)C
8d	O=C(N)C1=NC=C(C#CC2=CC(=CC=C2C)C(=O)NC3=CC=C(C(=C3)C(F)(F)F)CN4CCN(C)CC4)N1C
8e	O=C(NC1=CC=C(C(=C1)C(F)(F)F)CN2CCN(C)CC2)C3=CC=C(C(C#CC4=CN=C(C(=O)NC)N4C)=C3)C
8f	O=C(NC1=CC=C(C(=C1)C(F)(F)F)CN2CCN(C)CC2)C3=CC=C(C(C#CC4=CN=C(C(=O)NCCO)N4C)=C3)C
8g	O=C(N)C1=NC=C(C#CC2=CC(=CC=C2C)C(=O)NC3=CC=C(C(=C3)C(F)(F)F)CN4CCN(CCO)CC4)N1C
8h	O=C(NC1=CC=C(C(=C1)C(F)(F)F)CN2CCN(CCO)CC2)C3=CC=C(C(C#CC4=CN=C(C(=O)NC)N4C)=C3)C
8i	O=C(N)C1=NC=C(C#CC2=CC(=CC=C2C)C(=O)NC3=CC=C(C(=C3)C(F)(F)F)CN4CCN(CCOC)CC4)N1C
8j	O=C(N)C1=NC=C(C#CC2=CC(=CC=C2C)C(=O)NC3=CC=C(C(=C3)C(F)(F)F)CN4CCN(CC(=O)O)CC4)N1C
8k	O=C(NC1=CC=C(C(=C1)C(F)(F)F)CN2CCN(C)CC2)C3=CC=C(C(C#CC=4SC(=NC4)N)=C3)C
8l	O=C(NC1=CC=C(C(=C1)C(F)(F)F)CN2CCN(C)CC2)C3=CC=C(C(C#CC=4SC(=NC4)NC(=O)C)=C3)C
2a	C(#CC1=CN=C2C=CC=NN12)C=3C=C(C=CC3C)C4=NC5=CC=C(C=C5N4)CN6CCN(C)CC6
2b	C(#CC=1C=C(C=CC1C)C2=NC3=CC(=CC=C3N2)CN4CCN(C)CC4)C5=NN=C(C=C5)NC6CCC6
2c	C(#CC=1C=C(C=CC1C)C2=NC3=CC=C(C=C3N2)CN4CCN(C)CC4)C5=NN=C(C=C5)NC6CC6
2d	C(#CC=1C=C(C=CC1C)C2=NC3=CC=C(C=C3N2)CN4CCN(C)CC4)C5=NN=C(C=C5)NC(C)C
2e	C(#CC=1C=C(C=CC1C)C2=NC3=CC=C(C=C3N2)CN4CCN(C)CC4)C5=CN=C(N=C5)NC6CC6
2f	C(#CC1=CN=C2C=CC=NN12)C=3C=C(C=CC3C)C4=NC5=CC=C(C=C5N4)N6C=NC(=C6)C
2g	ClC=1C=CC(=CC1C#CC2=CN=C3C=CC=NN23)C4=NC5=CC=C(C=C5N4)CN6CCN(C)CC6
2h	ClC=1C=CC(=CC1C#CC2=NN=C(C=C2)NC3CCC3)C4=NC5=CC(=CC=C5N4)CN6CCN(C)CC6
2i	ClC=1C=CC(=CC1C#CC2=NN=C(C=C2)NC3CC3)C4=NC5=CC=C(C=C5N4)CN6CCN(C)CC6
2j	ClC=1C=CC(=CC1C#CC2=NN=C(C=C2)NC(C)C)C3=NC4=CC=C(C=C4N3)CN5CCN(C)CC5
2k	ClC=1C=CC(=CC1C#CC2=CN=C(N=C2)NC3CC3)C4=NC5=CC=C(C=C5N4)CN6CCN(C)CC6

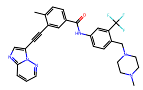

pona/CAS:943319-70-8

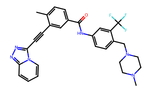

PF-114/CAS:1416241-23-0

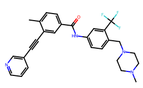

7a/CAS:1314640-64-6

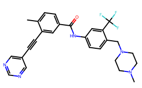

7b/CAS:1314640-65-7

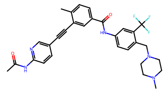

7c/CAS:1314640-66-8

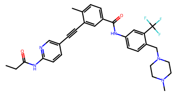

7d/CAS:1314640-67-9

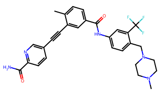

7e/CAS:1314640-68-0

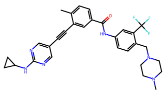

7f/CAS:1257628-62-8

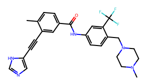

8a/CAS:1314640-69-1

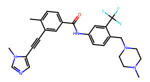

8b/CAS:1056942-46-1

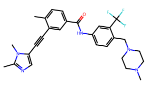

8c/CAS:1289555-63-0

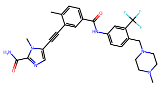

8d/CAS:957147-12-5

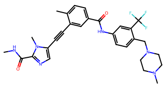

8e/CAS:1314640-70-4

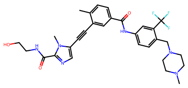

8f/CAS:1314640-71-5

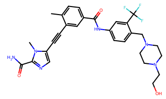

8g/CAS:1056942-51-8

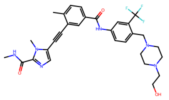

8h/CAS:1314640-72-6

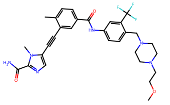

8i/CAS:1314640-73-7

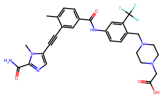

8j/CAS:1314640-75-9

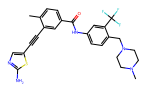

8k/CAS:1056942-55-2

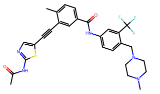

8l/CAS:1314640-76-0

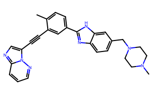

2a/CAS:1542265-12-2

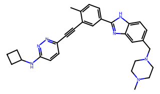

2b/CAS:1542265-10-0

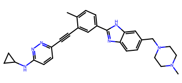

2c/CAS:1542265-11-1

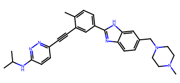

2d/CAS:1542265-13-3

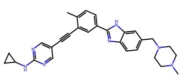

2e/CAS:1542265-14-4

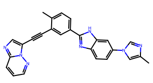

2f/CAS:1542265-20-2

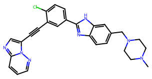

2g/CAS:1542265-15-5

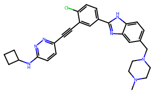

2h/CAS:1542265-17-7

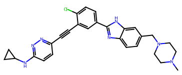

2i/CAS:1542265-16-6

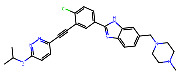

2j/CAS:1542265-18-8

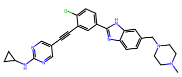

2k/CAS:1542265-19-9

Figure S1: 2D structures for TKIs dataset.

Table S9: SMILES for AChEIs dataset.

MOLECULE	SMILES
DNP	COC1=C(C=C2C(=C1)CC(C2=O)CC3CCN(CC3)CC4=CC=CC=C4)OC
3AD	OCCC1=C[C@H]2C[C@@H](C1)C=C(CCC(NC3=CC([C@@H]([NH2]CC#C)CC4)=C4C=C3)=O)C2
3AS	OCCC1=C[C@H]2C[C@@H](C1)C[C@H](CCC(NC3=CC([C@@H]([NH2+]CC#C)CC4)=C4C=C3)=O)C2
3CD	OCCC1=C[C@H]2C[C@@H](C1)C=C(CCC(C3=CC([C@H]([NH2]CC#C)CC4)=C4C=C3)=O)C2
3CS	OCCC1=C[C@H]2C[C@@H](C1)C[C@H](CCC(C3=CC([C@H]([NH2+]CC#C)CC4)=C4C=C3)=O)C2
3ED	OCCC1=C[C@H]2C[C@@H](C1)C=C(CCCOC3=CC([C@@H]([NH2]CC#C)CC4)=C4C=C3)C2
3ES	OCCC1=C[C@H]2C[C@@H](C1)C[C@H](CCCOC3=CC([C@@H]([NH2+]CC#C)CC4)=C4C=C3)C2
3HD	OCCC1=C[C@H]2C[C@@H](C1)C=C(CCCC3=CC([C@@H]([NH2]CC#C)CC4)=C4C=C3)C2
3HS	OCCC1=C[C@H]2C[C@@H](C1)C[C@H](CCCC3=CC([C@@H]([NH2+]CC#C)CC4)=C4C=C3)C2
3SD	OCCC1=C[C@H]2C[C@@H](C1)C=C(CCC(OC3=CC([C@@H]([NH2]CC#C)CC4)=C4C=C3)=O)C2
3SS	OCCC1=C[C@H]2C[C@@H](C1)C[C@H](CCC(OC3=CC([C@@H]([NH2+]CC#C)CC4)=C4C=C3)=O)C2
5AD	OCCC1=C[C@H]2CC(C1)C=C(CCCCC(NC3=CC=C4C([C@@H]([NH2+]CC#C)CC4)=C3)=O)C2
5AS	OCCC1=C[C@H]2C[C@@H](C1)C[C@H](CCCCC(NC3=CC=C4C([C@@H]([NH2+]CC#C)CC4)=C3)=O)C2
5CD	OCCC1=C[C@H]2CC(C1)C=C(CCCCC(C3=CC=C(CC[C@H]4[NH2+]CC#C)C4=C3)=O)C2
5CS	OCCC1=C[C@H]2C[C@@H](C1)C[C@H](CCCCC(C3=CC=C(CC[C@H]4[NH2+]CC#C)C4=C3)=O)C2
5ED	OCCC1=C[C@H]2CC(C1)C=C(CCCCCOC3=CC=C4C([C@@H]([NH2+]CC#C)CC4)=C3)C2
5ES	OCCC1=C[C@H]2C[C@@H](C1)C[C@H](CCCCCOC3=CC=C4C([C@@H]([NH2+]CC#C)CC4)=C3)C2
5HD	OCCC1=C[C@H]2CC(C1)C=C(CCCCCC3=CC=C(CC[C@H]4[NH2+]CC#C)C4=C3)C2
5HS	OCCC1=C[C@H]2C[C@@H](C1)C[C@H](CCCCCC3=CC([C@@H]([NH2+]CC#C)CC4)=C4C=C3)C2
5SD	OCCC1=C[C@H]2CC(C1)C=C(CCCCC(OC3=CC=C4C([C@@H]([NH2+]CC#C)CC4)=C3)=O)C2
5SS	OCCC1=C[C@H]2C[C@@H](C1)C[C@H](CCCCC(OC3=CC=C4C([C@@H]([NH2+]CC#C)CC4)=C3)=O)C2
SAD	OCCC1=C[C@@H]2C(CCC(NC3=CC([C@@H]([NH2+]CC#C)CC4)=C4C=C3)=O)=C[C@H]1C2
SAS	OCCC1=C[C@H]2[C@@H](CCC(NC3=CC([C@@H]([NH2+]CC#C)CC4)=C4C=C3)=O)C[C@@H]1C2
SCD	OCCC1=C[C@@H]2C(CCC(C3=CC([C@@H]([NH2+]CC#C)CC4)=C4C=C3)=O)=C[C@H]1C2
SCS	OCCC1=C[C@H]2[C@@H](CCC(C3=CC([C@@H]([NH2+]CC#C)CC4)=C4C=C3)=O)C[C@@H]1C2
SED	OCCC1=C[C@@H]2C(CCC(OC3=CC([C@@H]([NH2+]CC#C)CC4)=C4C=C3)=O)=C[C@H]1C2
SES	OCCC1=C[C@@H]2[C@@H](CCCOC3=CC([C@@H]([NH2+]CC#C)CC4)=C4C=C3)C[C@H]1C2
SHD	OCCC1=C[C@H]2C(CCCC3=CC([C@@H]([NH2+]CC#C)CC4)=C4C=C3)=C[C@@H]1C2
SHS	OCCC1=C[C@@H]2[C@@H](CCCC3=CC([C@@H]([NH2+]CC#C)CC4)=C4C=C3)C[C@H]1C2
SSD	OCCC1=C[C@H]2C(CCCOC3=CC([C@@H]([NH2+]CC#C)CC4)=C4C=C3)=C[C@@H]1C2
SSS	OCCC1=C[C@H]2[C@H](C[C@@H]1C2)CCC(OC3=CC4=C(C=C3)CC[C@@H]4[NH2+]CC#C)=O

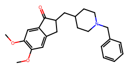

DNP

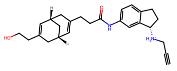

3AD

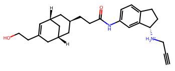

3AS

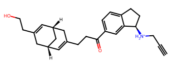

3CD

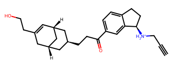

3CS

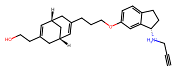

3ED

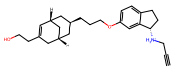

3ES

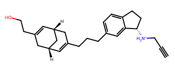

3HD

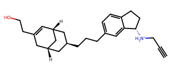

3HS

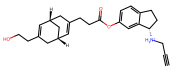

3SD

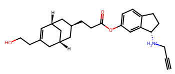

3SS

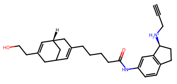

5AD

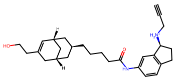

5AS

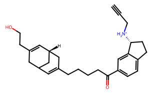

5CD

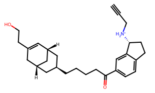

5CS

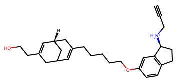

5ED

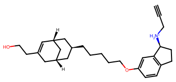

5ES

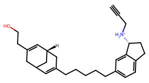

5HD

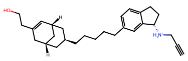

5HS

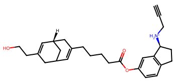

5SD

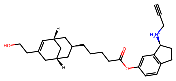

5SS

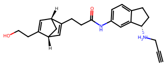

SAD

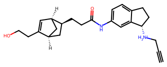

SAS

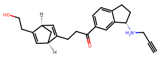

SCD

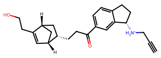

SCS

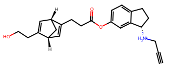

SED

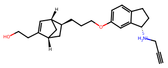

SES

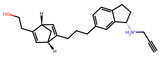

SHD

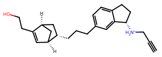

SHS

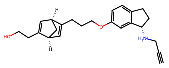

SSD

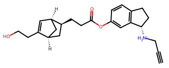

SSS

Figure S2: 2D structures for AChEIs based huprine-ladostigil heterodimers dataset.

Table S10: SMILES of potential heterodimers AChEIs dataset.

MOLECULE	SMILES
THA	NC1=C2CCCCC2=NC2=CC=CC=C12
THC	[H][C@@]12C=C(C)CC[C@@]1([H])C(C)(C)OC1=C2C(O)=CC(CCCCC)=C1
CBDA	Oc1c([C@H]2[C@@H](CCC(=C2)C)C(=C)C)c(O)cc(c1C(=O)O)CCCCC
CBDA0	CC(=C)[C@@H]1CCC(C)=C[C@H]1c1c(O)cc(CCCCCCNC2=C3CC=CC=C3NC3=C2CCCC3)c(C(O)=O)c1O
CBDA1	CC(=C)[C@@H]1CCC(C)=C[C@H]1c1c(O)cc(CCCCCC(=O)NC2=C3C=CC=CC3NC3=C2CCCC3)c(C(O)=O)c1O
CBDA2	CC(=C)[C@@H]1CCC(C)=C[C@H]1c1c(O)cc(CCCC(=O)CCNC2=C3CC=CC=C3NC3=C2CCCC3)c(C(O)=O)c1O
CBDA3	CC(=C)[C@@H]1CCC(C)=C[C@H]1c1c(O)cc(CC(=O)CCCCNC2=C3CC=CC=C3NC3=C2CCCC3)c(C(O)=O)c1O
F	CC1=C2CCC=C[C@H]2c2ccc(CCNC(=O)CC3=C4C=CC=CC4=[NH+]C4=C3CCCC4)cc2O1
THC0	CC1=C[C@@H]2[C@@H](CC1)C(C)(C)Oc1cc(CCCCCCNC3=C4C=CC=CC4NC4=C3CCCC4)cc(O)c21
THC1	CC1=C[C@@H]2[C@@H](CC1)C(C)(C)Oc1cc(CCCCCC(=O)NC3=C4C=CC=CC4NC4=C3CCCC4)cc(O)c21
THC2	CC1=C[C@@H]2[C@@H](CC1)C(C)(C)Oc1cc(CCCC(=O)CCNC3=C4C=CC=CC4NC4=C3CCCC4)cc(O)c21
THC3	CC1=C[C@@H]2[C@@H](CC1)C(C)(C)Oc1cc(CC(=O)CCCCNC3=C4C=CC=CC4NC4=C3CCCC4)cc(O)c21

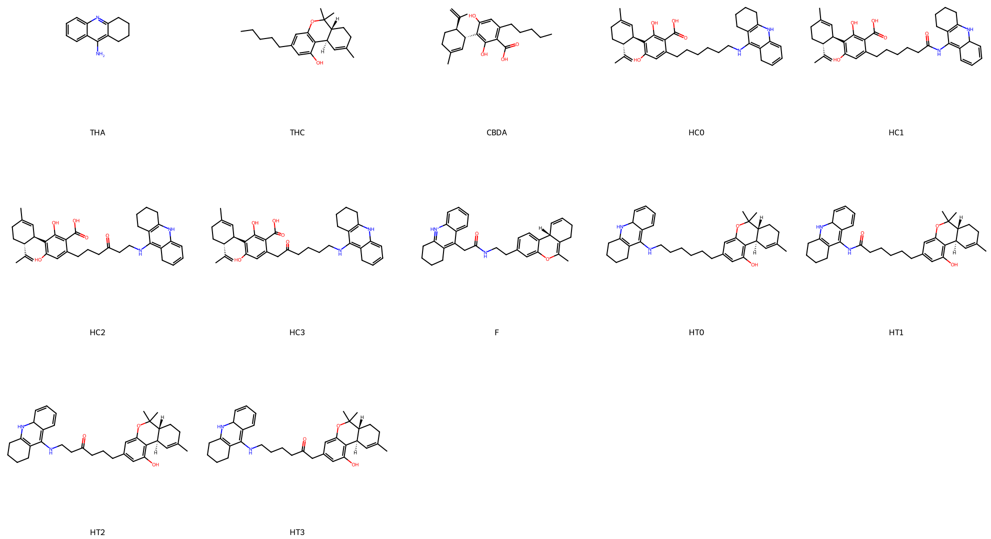

Figure S3: 2D structures for AChEIs based tacrine-thc-cbda heterodimers dataset.

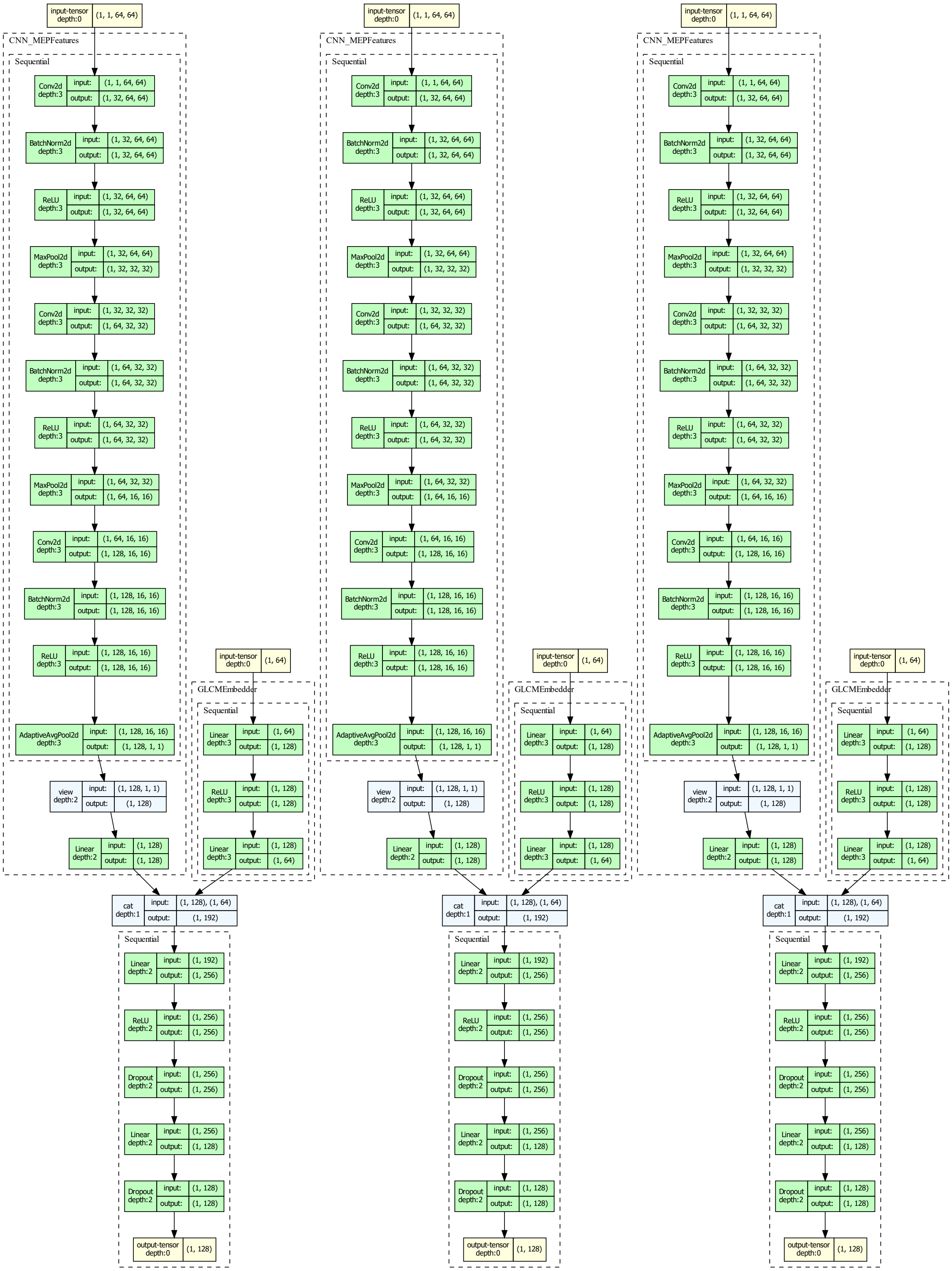

Figure S4: Architecture of the developed model

Architecture and Implementation Details

The model architecture receives an input set $\mathbf{X}_{MEP} \subseteq \mathbb{R}^{1\times\text{height}\times\text{width}}$ for the MEP images, and $\mathbf{X}glcm \leftarrow rdg \subseteq \mathbb{R}^{\mathrm{d}_t}$ for the GLCM matrices generated from the corresponding RDG diagram images, with $\mathrm{d}_t = 64$, given that the GLCM matrices were defined with 8 levels (8 x 8). The model architecture is divided into three parts; the first extracts visual information from the MEP images.

$$f_{\text{cnn}} : \mathbf{X}_{MEP} \to \mathbb{R}^{\mathrm{d_c}},\ f_{\text{cnn}}(\mathbf{x}_{MEP}) = \mathbf{W}_{\mathrm{c}} \cdot \mathit{Flatten}(\mathrm{CNN}(\mathbf{x}_{MEP})) + \mathrm{b_c} \tag{1}$$

Where $\mathrm{d_c} = 128$ is the dimension, $\mathbf{W}\mathrm{c}$ is the weight vector of the last linear layer of the CNN submodel, which consists of three blocks of Conv2D + ReLU + Pooling + AdaptiveAvgPool (further details in Figure 4 in the supplementary material), and $\mathrm{b_c}$ is the bias of the last linear layer.

The second part of the model consists of a multilayer perceptron (MLP) with two linear layers, which learns texture information from the RDG diagrams corresponding to the MEP images, using the GLCM co-occurrence matrix as input.

$$f_{\text{mlp}} : \mathbf{X}_{glcm\leftarrow rdg} \to \mathbb{R}^{\mathrm{d_t}},\ f_{\text{mlp}}(\mathbf{x}_{glcm\leftarrow rdg}) = \mathbf{W}_2 \cdot (\mathrm{ReLU}(\mathbf{W}_1 \cdot \mathbf{x}_{glcm\leftarrow rdg} + \mathrm{b}_1)) + \mathrm{b}_2 \tag{2}$$

Where $\mathrm{d_t} = 64$, $\mathbf{W}_1 \in \mathbb{R}^{128\times 64}$ and $\mathbf{W}_2 \in \mathbb{R}^{64\times 128}$. The third part of the model is a multimodal projector that combines the visual representations learned by the CNN in the first part with the texture features extracted by the MLP in the second part into a shared embedding space $\mathbb{R}^{\mathrm{d_e}}$ with $\mathrm{d_e} = 128$.

$$\phi : \mathbb{R}^{\mathrm{d}_\nu} \to \mathbb{R}^{\mathrm{d_e}},\ \phi(\nu) = \mathit{Dropout}(\mathbf{W}_4 \cdot \mathit{Dropout}(\mathrm{ReLU}(\mathbf{W}_3 \cdot \nu + \mathrm{b}_3)) + \mathrm{b}_4) \tag{3}$$

Where $\mathbf{W}_3 \in \mathbb{R}^{128\times 192}$, $\mathbf{W}_4 \in \mathbb{R}^{128\times 128}$ and $\mathrm{d}_\nu = 192$ is the concatenated dimension resulting from $\mathrm{d_c}$ and $\mathrm{d_t}$, ν is the vector in the concatenated space, and the dropout prob-

ability is set to 0.3 for the *Dropout*() function. Therefore, the global embedding function $\Phi(\mathbf{x}_{\mathrm{MEP}}, \mathbf{x}_{\mathrm{glcm \leftarrow rdg}})$ can be defined as

$$\Phi : \mathbb{R}^{\mathrm{d_c}} \times \mathbb{R}^{\mathrm{d_t}} \rightarrow \mathbb{R}^{\mathrm{d^e}},\ \Phi(\mathbf{x}_{\mathrm{MEP}}, \mathbf{x}_{\mathrm{glcm \leftarrow rdg}}) = \phi([f_{\mathrm{cnn}}(\mathbf{x}_{\mathrm{MEP}}); f_{\mathrm{glcm \leftarrow rdg}}(\mathbf{x}_{\mathrm{glcm \leftarrow rdg}})]) \quad (4)$$

The cost function that the model aims to maximize is the triplet loss, as it is a Siamese network designed to learn similarity and dissimilarity between images.

$$\mathcal{L} = \max(0, ||\Phi(\mathrm{a}_{MEP}, \mathrm{a}_{\mathrm{glcm \leftarrow rdg}}) - \Phi(\mathrm{p}_{MEP}, \mathrm{p}_{\mathrm{glcm \leftarrow rdg}})||_2^2 - ||\Phi(\mathrm{a}_{MEP}, \mathrm{a}_{\mathrm{glcm \leftarrow rdg}}) - \Phi(\mathrm{n}_{MEP}, \mathrm{n}_{\mathrm{glcm \leftarrow rdg}})||_2^2 + \alpha) \quad (5)$$

Where α is the margin that defines the minimum distance between the positive and negative pair distances, a_{MEP} and $\mathrm{a}_{glcm \leftarrow rdg}$ are the anchor values, p_{MEP} and $\mathrm{p}_{glcm \leftarrow rdg}$ correspond to the positive samples (similar images), and n_{MEP} and $\mathrm{n}_{glcm \leftarrow rdg}$ correspond to the negative samples (images dissimilar to the anchor). Finally, $||\cdot||_2$ denotes the Euclidean distance (L2 norm). The algorithm below presents the developed method using simplified mathematical notation.

Algorithm: Training of the Siamese Network for Molecular Similarity via MEP and GLCM

Require: Set of MEP images and GLCM vectors, similarity matrix $\mathrm{S_T}$, margin α, number of epochs E, and model Φ

Initialize CNN, MLP, and the multimodal projector of Φ
for $\mathrm{e} = 1$ **to** E **do** ▷ For each epoch
  Build triplets $(\mathrm{a}, \mathrm{p}, \mathrm{n})$ such that:
  $\mathrm{S}_T(\mathrm{a}, \mathrm{p}) \geq 0.70$ (positive) and $\mathrm{S}_T(\mathrm{a}, \mathrm{n}) \leq 0.40$ (negative) ▷ Or another criterion
  for each triplet $(\mathrm{a}, \mathrm{p}, \mathrm{n})$ **do**
    $\mathrm{z_a} \leftarrow \Phi(\mathrm{a}_{MEP}, \mathrm{a}_{glcm})$
    $\mathrm{z_p} \leftarrow \Phi(\mathrm{p}_{MEP}, \mathrm{p}_{glcm})$
    $\mathrm{z_n} \leftarrow \Phi(\mathrm{n}_{MEP}, \mathrm{n}_{glcm})$
    Compute loss:
  $\mathcal{L} \leftarrow \max(0, \|\mathrm{z_a} - \mathrm{z_p}\|^2 - \|\mathrm{z_a} - \mathrm{z_n}\|^2 + \alpha)$
    Update weights via backpropagation using $\mathcal{L}$
  end for
end for
return Φ
